# Androgen Receptor Splice Variants Contribute to the Upregulation of DNA Repair in Prostate Cancer

**DOI:** 10.3390/cancers14184441

**Published:** 2022-09-13

**Authors:** Yuri Tolkach, Anika Kremer, Gábor Lotz, Matthias Schmid, Thomas Mayr, Sarah Förster, Stephan Garbe, Sana Hosni, Marcus V. Cronauer, Ildikó Kocsmár, Éva Kocsmár, Péter Riesz, Abdullah Alajati, Manuel Ritter, Jörg Ellinger, Carsten-Henning Ohlmann, Glen Kristiansen

**Affiliations:** 1 Institute of Pathology, University Hospital Bonn, 53127 Bonn, Germany; 2 Institute of Pathology, University Hospital Cologne, 50937 Cologne, Germany; 3 Department of Pathology, Forensic and Insurance Medicine, Semmelweis University, 1085 Budapest, Hungary; 4 Department of Medical Biometry, Informatics, and Epidemiology (IMBIE), University Hospital Bonn, 53127 Bonn, Germany; 5 Department of Radiation Oncology, University Hospital Bonn, 53127 Bonn, Germany; 6 Clinic of Urology, University Hospital Bonn, 53127 Bonn, Germany; 7 Department of Urology, Semmelweis University, 1085 Budapest, Hungary; 8 Department of Urology, Johanniter Krankenhaus Bonn, 53113 Bonn, Germany

**Keywords:** prostate cancer, DNA repair, BRCA1, BRCA2, androgen receptor, splice variant, AR-V7, castration-refractory prostate cancer, androgen deprivation therapy

## Abstract

**Simple Summary:**

Ligand-independent androgen receptor splice variants emerge during androgen deprivation therapy and are suspected to render prostate carcinomas castration-resistant. In a retrospective analysis of a large cohort of primary and advanced prostate tumors, we observed increased expression of androgen receptor splice variants in therapy refractory tumors. Our hypothesis was that AR splice variants exert their tumor-promoting activity by modulating the intrinsic DNA repair machinery. In the sequence from primary over advanced tumors under androgen-deprivation therapy to castration resistance, AR splice variant expression increases and is linked to increased expression of DNA repair genes. This effect of AR splice variants appeared independent of their known impact on tumor cell proliferation. These clinical findings were validated in an androgen-sensitive prostate cancer cell line that mimics a castration-resistant phenotype by overexpression of AR-V7. Modulated DNA repair gene expression in the presence of AR splice variants is linked to increased DNA repair activity, pointing at a novel therapeutic approach for castration-resistant prostate cancer.

**Abstract:**

Background: Canonical androgen receptor (AR) signaling regulates a network of DNA repair genes in prostate cancer (PCA). Experimental and clinical evidence indicates that androgen deprivation not only suppresses DNA repair activity but is often synthetically lethal in combination with PARP inhibition. The present study aimed to elucidate the impact of AR splice variants (AR-Vs), occurring in advanced or late-stage PCA, on DNA repair machinery. Methods: Two hundred and seventy-three tissue samples were analyzed, including primary hormone-naïve PCA, primary metastases, hormone-sensitive PCA on androgen deprivation therapy (ADT) and castration refractory PCA (CRPC group). The transcript levels of the target genes were profiled using the nCounter platform. Experimental support for the findings was gained in AR/AR-V7-expressing LNCaP cells subjected to ionizing radiation. Results: AR-Vs were present in half of hormone-sensitive PCAs on androgen deprivation therapy (ADT) and two-thirds of CRPC samples. The presence of AR-Vs is highly correlated with increased activity in the AR pathway and DNA repair gene expression. In AR-V-expressing CRPC, the DNA repair score increased by 2.5-fold as compared to AR-V-negative samples. Enhanced DNA repair and the deregulation of DNA repair genes by AR-V7 supported the clinical data in a cell line model. Conclusions: The expression of AR splice variants such as AR-V7 in PCA patients following ADT might be a reason for reduced or absent therapy effects in patients on additional PARP inhibition due to the modulation of DNA repair gene expression. Consequently, AR-Vs should be further studied as predictive biomarkers for therapy response in this setting.

## 1. Introduction

Androgen receptor (AR) signaling and DNA repair are tightly interconnected in prostate cancer (PCA) [1,2,3,4,5,6,7,8]. The presence of pathogenic mutations in genes responsible for homologous recombination (HR) DNA repair opens up the possibility of therapy with PARP inhibitors (PARPi) in up to 25% of patients with metastatic castration-refractory PCA (CRPC) [9,10]. In cases of insufficiency in HR DNA repair, PARP is a reserve system that operates through base excision. Blocking base excision repair with PARPi renders tumor cells incapable of effectively repairing DNA damage, which then eventually accumulates lethal mutations [5,6]. However, there is early evidence that PARPi might be effective in CRPC in the absence of HR DNA gene mutations [5,6,11]. Androgen deprivation therapy (ADT) on PCA cells induces a so-called functional “BRCAness”. The term BRCAness defines an insufficiency in the HR DNA repair system [2,5,6], originally caused by the functional loss of the BRCA1 and BRCA2 genes. BRCA-deficient cells use error-prone DDR pathways that consequently increase their genome instability [12]. ADT treatment mimics this loss of BRCA1/2 expression. In this situation, PARPi deepens the DNA repair insufficiency, resulting in synthetic lethality for tumor cells. It has also been shown in vitro that both *PARP1* and *PARP2* are critical effectors of the AR pathway activity. Besides their function in DNA repair, PARP enzymes are known to be transcriptional coactivators of the AR. Notably, PARP-1 appears to activate AR function and affect downstream signaling [13,14], which is another rationale for targeting PARP in PCA [5,6,7,8,15,16].

The synthetic lethality of ADT and PARPi might, however, be dependent on AR alterations (splice variants, amplification, mutations) often present in advanced PCA [17,18,19,20] and responsible for sustained AR pathway activity during ADT. The upregulation of AR-V7 in clinical samples of advanced PCA patients was reported by Sharp et al. [21]. DNA repair in PCA cell lines exposed to ionizing radiation was found to be diminished following AR blockade with enzalutamide but largely preserved in the case of AR-V7 and ARv567es splice variant expression [3]. There is limited evidence that both full-length AR (AR-FL) and AR splice variants (AR-Vs) directly activate the expression of key genes necessary for DNA repair [2,3,8,22]. Apparently, both non-homologous end-joining and HR DNA repair are the main effectors of AR-FL and AR-Vs in prostate cancer [1,2,4]. Blocking AR-FL using antiandrogens has been shown to retain AR-V activity with regard to supporting the DNA repair system. However, so far, this has mainly been shown in cell line models [3,8,23].

The present study aimed to clarify, in clinical PCA samples, whether the expression of key DNA repair genes is affected by ADT, particularly in the presence of AR-Vs. A cohort of patients in different stages of PCA was analyzed to characterize AR pathway activity, and these data were correlated with the expression of DNA repair genes. Luo et al. reported that DNA repair is modulated by androgen receptor splice variant 7 [23]. The present study provides evidence that DNA repair is partly dependent on AR pathway activity in PCA. As enhanced DNA repair is induced in the presence of AR-Vs through the modulation of DNA repair gene expression, the application of synthetic lethality concepts, such as the combination of ADT and PARPi, might be questionable.

## 2. Materials and Methods

### 2.1. Patient Cohort

The study cohort consisted of 184 patients: 167 patients with PCA in different disease stages and 17 patients from control groups (Table 1). All patients received ADT (LHRH analogs/antagonists) alone or in combination with abiraterone or antiandrogens (bicalutamide, etc.). Two patients with primary small-cell carcinoma of the prostate and sarcomatous carcinoma lacking prostate epithelial differentiation after ADT were also included. Materials on twenty-two patients with CRPC were provided by the Department of Pathology, Forensic and Insurance Medicine, Semmelweis University, Budapest, Hungary (2005–2016). All other patients were diagnosed at the Institute of Pathology of the University Hospital Bonn, Bonn, Germany (2003–2018).

### 2.2. Samples

**Figure 1:**
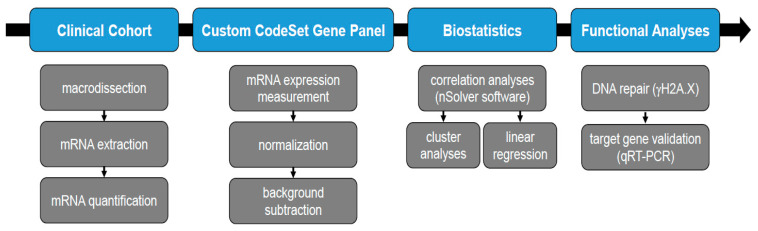
Flow chart of the main steps in this study: material processing, data acquisition, biostatistical analyses and experimental validation.

**Figure 2:**
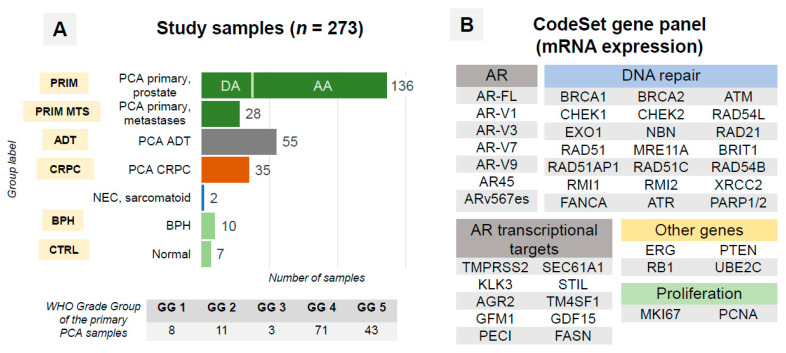
(**A**) Structure of the samples in study cohort and group labels used in further Figures. Among primary prostate cancer samples, 33 ductal adenocarcinoma (DA) samples were analyzed. Abbreviations: AA—acinar adenocarcinoma, ADT—androgen deprivation therapy (primary tumor and metastases), BPH—benign prostatic hyperplasia, CRPC—castration-refractory prostate cancer (primary tumor and metastases), CTRL—normal prostate tissue, GG—grade group, NEC—neuroendocrine carcinoma, PCA—prostate cancer, WHO—World Health Organization. (**B**) Composition of the gene panel for mRNA expression analysis using nCounter technology. AR—androgen receptor, FL—full-length. Additionally, four housekeeping genes (HPRT1, ALAS1, ARF1, PGK1) were included in the panel.

All samples were harvested from formalin-fixed paraffin-embedded (FFPE) tissue blocks (flow chart: see Figure 1). Multiple samples (up to 4) from tumors of selected patients were analyzed to address heterogeneity of primary and metastatic tumors with an overall number of 273 samples passing quality control (Figure 2A). All tumor samples had a purity of >90% tumor cells and were macrodissected. Among included CRPC samples, 29 were from primary tumor (no treatment with curative intent) and 6 from metastatic lesions (2 from bone and 2 from soft tissue metastases, 1 from liver and 1 from retroperitoneal lymph node).

### 2.3. RNA Extraction

One or several 10 μm sections from paraffin block were used for macrodissection and total mRNA extraction. PureLink™ FFPE RNA Isolation Kit (ThermoFisher Scientific, Waltham, MA, USA) was used according to the manufacturer’s instructions. NanoDrop 2000 spectrophotometer (Thermo Scientific, USA) was applied for mRNA quality control and quantification.

### 2.4. RNA Expression Analysis

All RNA expression analyses were performed using the nCounter platform (NanoString Technologies, Inc.; Seattle, WA, USA). A custom CodeSet gene panel (Supplementary Table S1) included 45 target genes: (1) AR-FL and splice variants (AR-Vs; junction-specific probes), (2) AR transcriptional targets, (3) DNA repair-associated genes, (4) proliferation-related genes, and (5) further genes relevant for PCA (Figure 2B). Four housekeeping genes (*HPRT1, ALAS1, ARF1, PGK1*) were included. All samples were titrated to 100 ng of the total mRNA amount. Internal nCounter negative and positive controls as well as other internal metrics (RNA quantity, binding density) were used for quality control.

### 2.5. Statistical Analysis

Raw RNA expression data were processed by nSolver Analysis Software v. 4.0.70 (Nanostring Technologies, Seattle, WA, USA). Negative controls were used for background subtraction (geometric mean) of the called expression values. Internal positive controls and reference genes were used for normalization of expression levels. All further analyses were carried out in R (v. 3.6.0, The R Foundation for Statistical Computing). AR pathway score (AR score) was calculated from 10 genes representing transcriptional targets of AR (Figure 2B), as described in [19], with benign non-hyperplastic prostatic tissue used as reference. In brief, for each gene, a z-score was calculated by subtracting the pooled mean of expression in reference tissue samples divided by the pooled standard deviation of expression in reference tissue samples. AR score output was calculated as the sum of z-scores for all ten target genes divided by the number of genes. The HRDNA repair score (DNA-R score) was calculated using the same principle using all genes (*n* = 20) from our panel related to HR DNA repair (excluding *PARP1* and *PARP2*). The expression of AR-Vs was evaluated both quantitatively (correlation analyses, heatmap) and qualitatively. For qualitative estimation of AR-Vs (present/not present), a threshold of 20 normalized counts (approximately 5 standard deviations above mean AR-V expression for control tissue samples) was used after background subtraction of the geometric mean of negative control samples. Appropriate parametric (*t*-test), non-parametric (Mann–Whitney U test) and correlation statistical tests (Pearson’s *r*) were used for comparison between groups and parameters. Expression heatmaps were created in nSolver 4.0 analysis software (nanoString, Seattle, WA, USA) using Pearson’s correlation for clustering.

### 2.6. Linear Regression Analysis

To analyze the joint effects of AR-Vs and proliferation on DNA repair, score variables that quantified either AR-Vs or the proliferation of tumor cells were constructed. These scores were set up in the same way as the DNA-R score using z-scores of the AR splice variants (AR-V1, -V3, -V7, -V9) and proliferation genes (MKI67, PCNA) for both the splice variant (SV score) and proliferation (P score) scores, respectively. Values of multiple samples from single patients were averaged. In the next step, the scores were used to fit linear regression models with the DNA-R score as the dependent variable and the SV and P scores as independent variables. The fits of the linear regression models were used to investigate whether AR-Vs had an effect on DNA repair when accounting for the effect of tumor cell proliferation on DNA repair (Supplementary Figure S9).

### 2.7. Cell Culture and Irradiation

LNCaP cells were obtained from the German Collection of Microorganisms and Cell Cultures (Braunschweig, Germany). LNCaP cells stably overexpressing AR-FL (LNCaP/AR) or AR-V7 (LNCaP/V7) were generated by lentiviral transduction. Lentivirus was generated in HEK 293T cells via co-transfection of VSV-G, Gag-Pol and AR-FL/ V7 expressing plasmids using jetPRIME (Polyplus, Illkirch, France). pLENTI6.3/AR-GC-E2325 (lentiviral vector for AR-FL expression [24]) was a gift from Karl-Henning Kalland, and AR-V7-pcw107, described in Martz et al., 2014 [25], was a gift from David Sabatini and Kris Wood. LNCaP/AR cells were selected with 5.5 µg/mL Blasticidin (Life Technologies, Paisley, UK) for 2 weeks, and LNCaP/V7 cells were selected with 1 µg/mL Puromycin (Cayman Chemical, Ann Arbor, USA) for 5 days.

Cells were cultured in RPMI1640 medium containing GlutaMax supplemented with 1% penicillin/streptomycin (10,000 U/mL) and 10% fetal bovine serum (Life Technologies, Paisley, UK) or 10% charcoal-stripped fetal bovine serum (DCC) (Biowest, Nuaillé, France) at 37 °C in a humidified atmosphere containing 5% CO_2_. Cells were routinely checked to exclude mycoplasma contamination.

Radiation experiments were performed on a linear accelerator (Truebeam Stx, Varian Medical System, Palo Alto, CA, USA) using 6 MeV photon energy at a dose rate of 4 Gy/min at dose maximum (D_max_ = 20 mm). Depending on the experimental setting, the applied dose to the cells varied between 2 to 6 Gy. For this purpose, cells seeded in 6-well plates (for Western Blot and RNA analysis) or on glass coverslips placed in 12-well plates (for IF) were positioned in a tissue-equivalent RW3-plasticphantom (PTW, Freiburg, Germany) at a depth of D_max_. The field size was adapted according to the number of plates irradiated.

### 2.8. γH2A.X Assay

DNA double-strand breaks were determined by γH2A.X staining cells [26]. Cells were grown for 24 h in androgen-deprived medium and then subjected to 2 Gy irradiation. Immunofluorescence was performed 24 h post-irradiation, as previously described [27]. Foci were visualized using phospho-Histone H2A.X (catalog number 05-636; Millipore, Temecula, CA, USA) and anti-mouse IgG/IgM Alexa Fluor 488-conjugated secondary antibodies (Dianova, Hamburg, Germany). Subsequently, cells were embedded in Fluoromount-G with DAPI (Life Technologies, Carlsbad, CA, USA). Fluorescent images were acquired on an Olympus CKX53 microscope (Tokyo, Japan) and foci were counted using QuPath Software v0.3.2 [28]. At least 200 cells per condition were counted.

### 2.9. Immunoblot

Cell lysates were prepared using RIPA lysis buffer supplemented with protease and phosphatase inhibitors (Complete Protease Inhibitor Cocktail, Roche, Basel, Switzerland and Halt Protease and Phosphatase Inhibitor Thermo Scientific, Rockford, IL, USA) as previously described [29]. Primary antibodies used included AR-V7 (31-1109-00, RevMab Biosciences, San Francisco, CA, USA), γH2A.X (05-636, Merck Millipore, Darmstadt, Germany), and β-Actin (ab6276, Abcam, Cambridge, UK). As a secondary antibody, a horseradish peroxidase-conjugated antibody (ab6789, Abcam) was used. Signals were detected using ECL Western Blot Substrate or SuperSignal West Dura Extended Duration Substrate (Thermo Scientific, Rockford, IL, USA) on a Fusion S imaging system (Vilber Lourmat, Radolfzell, Germany).

### 2.10. Quantitative qRT-PCR

To study the mRNA expression of DNA repair genes (Supplementary Table S1) in LNCaP cells stably overexpressing AR-FL or AR-V7, cells were seeded 24 h prior to irradiation in androgen-deprived medium. Six hours after irradiation (6Gy), either protein lysates or total RNA were recovered from the cells, followed by qRT-PCR as previously described [29]. Oligonucleotide primers specific for DNA repair genes and PPIA (peptidyl-prolyl isomerase A, used as housekeeping gene) were purchased from biomers.net (Ulm, Germany). Primer sequences are provided in Supplementary Table S2. Sequence verification of the amplification products was performed with Sanger sequencing. Gene expression was measured in triplicates per gene. Relative gene expression was assessed using the ΔΔC_τ_ method with PPIA as a housekeeping gene (Supplementary Tables S3 and S4).

### 2.11. Ethical Considerations

The study was approved by the ethical committees of the University of Bonn (Votum 124/19) and Semmelweis University (#177/2016).

## 3. Results

### 3.1. Quality Control (QC)

Three and five samples were excluded at the QC stage from the ADT and CRPC groups, respectively, due to low RNA quality (the final composition is in Table 1, excluding samples failing QC). The excluded samples were small, decalcified bone biopsies and transurethral resections. 

### 3.2. AR-Vs Appear Mostly as a Response to ADT

**Figure 3:**
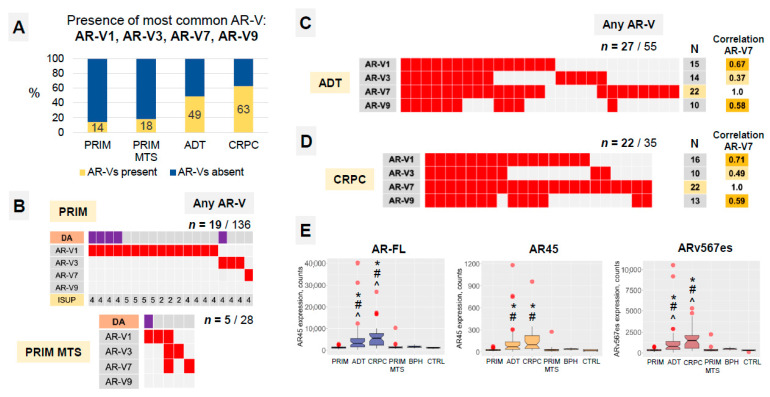
(**A**) Analysis of samples expressing any of the four androgen receptor splice variants in the four main study groups. BPH and CTRL groups were negative for AR-Vs. Numbers represent percentages of samples containing AR-Vs. (**B**–**D**) Stratification of the samples expressing any of the AR-V splice variants (V1, V3, V7, V9) in study groups. Boxes represent single samples. Stratification according to histological subtype (acinar or ductal (DA) adenocarcinoma) in primary hormone-naïve tumor samples. As other AR-Vs are co-expressed with AR-V7 in CRPCs, the latter appears a reasonable surrogate marker for the presence of AR-Vs. Correlation (co-existence) measure is presented for AR-V1, -V3 and -V9 compared to AR-V7 splice variant. “*n*” represents the number of samples positive for single AR splice variants. (**E**) Expression of full-length AR (AR-FL) and two other splice variants (AR45 and ARv567es) in tumor and benign study groups. Statistical significance (*p* < 0.05): * vs. PRIM group, ^ CRPC vs. ADT group, # vs. CTRL group (Mann–Whitney U-test).

An analysis of AR-FL and AR-V mRNA expression (AR-V1, AR-V3, AR-V7, AR-V9, AR45, ARv567es) was performed on the nCounter platform (Figure 1 and Figure 3). AR-Vs were detected in approximately two-thirds of CRPC samples and half of ADT samples but in only 14% of hormone-naïve cases (84% with Gleason Score > 4 + 3; no statistical association with ductal/acinar morphology). We detected no expression of AR-Vs in normal tissue and BPH samples (Figure 3A,B). AR-V1 was the most common AR-V in hormone-naïve PCA. AR-V7 was most common during ADT and in the CRPC stage (Figure 3B–D). In fact, in CRPC samples, AR-V7 was present in 100% of the samples expressing any of the other AR-Vs (Figure 3D). One of two CRPC patients on abiraterone therapy in our cohort expressed AR-Vs.

Both AR45 and ARv567es mRNA were detectable in tumor and benign samples (Figure 3E). AR-FL, AR45 and ARv567es mRNA expressions were significantly higher in ADT and CRPC samples (all *p* < 0.001). AR45 and ARv567es mRNA expressions were highly correlated with the expression of AR-FL (Pearson’s *r* 0.86 and 0.99, respectively, both *p* < 1.0 × 10^−10^). ARv567es mRNA expression was approximately three and four times higher (both *p* < 0.0001) in presence of any of the other AR-V splice variants in the CRPC and ADT groups, correspondingly. 

A certain level of intra-patient heterogeneity was evident regarding the presence of AR-Vs. The AR-V status of multiple samples from single patients was heterogeneous in 9 out of 41 patients in the PRIM group, 1 of 4 patients in PRIM MTS, and 3 of 10 patients in the ADT group, but not in the CRPC group.

Both samples with primary small cell neuroendocrine carcinoma of the prostate and sarcomatoid carcinoma (post-ADT) showed no or almost undetectable expression levels of AR-Vs and AR-FL, respectively [30].

### 3.3. AR Signaling and Proliferation Depend on the Presence of AR-Vs in ADT and CRPC Tumors

To measure the activation of the AR pathway, we calculated an AR score from cumulative levels of expression for 10 established transcriptional targets of AR (Figure 2B) with CTRL samples as a reference. 

**Figure 4:**
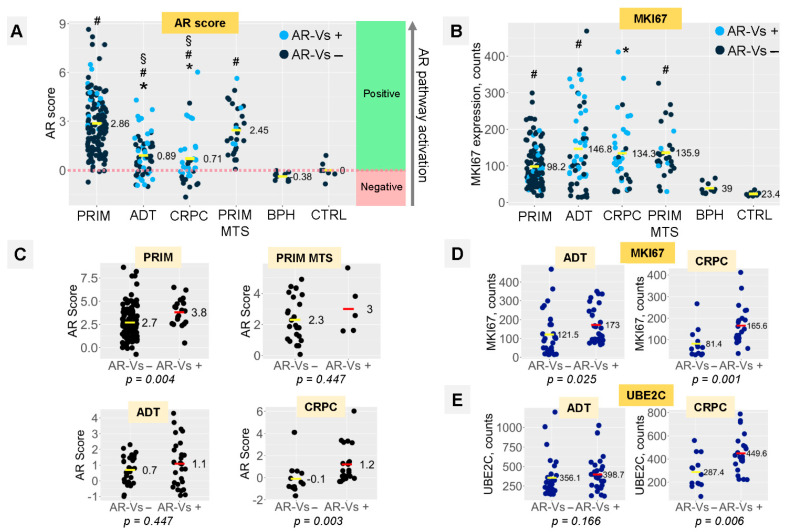
(**A**) Distribution of AR scores in study groups (cumulative score of AR pathway activation, calculated based on mRNA expression of 10 transcriptional targets of AR). Light blue points express any of the AR-V splice variants (V1, V3, V7, V9); dark blue points do not express AR-V splice variants. “Positive” area represents AR score in “activated” range compared to reference group (CTRL: benign non-hyperplastic prostate tissue). “Negative” area represents depression in AR signaling. (**B**) mRNA expression of proliferation marker MKI67. (**C**) Analysis of AR score distribution in four “tumor” study groups in relation to dependence on AR-V splice variant expression. (**D**) Analysis of MKI67 mRNA expression in ADT and CRPC groups in relation to dependence on AR-V splice variant expression. (**E**) Analysis of UBE2C mRNA expression in ADT and CRPC groups in relation to dependence on AR-V splice variant expression. *p*-levels calculated using Mann–Whitney U-test. Statistical significance (*p* < 0.05): * vs. PRIM group, # vs. CTRL group, § vs. PRIM MTS group.

The median AR-FL expression increased with the progression of the disease (Figure 3E). However, AR target gene (positive AR score) induction was significantly reduced in the ADT and CRPC samples (despite ongoing ADT), while AR signaling was significantly activated in samples from both the PRIM and PRIM MTS groups (Figure 4A). In fact, a smaller part of the ADT and CRPC samples revealed the downregulation of known AR target genes (negative AR score) (Figure 4A).

The decrease in the AR score, as observed for ADT and CRPC, however, does not translate into a significant change in the proliferative index. Similar to the PRIM MTS group, MKI67 expression was significantly elevated in ADT and CRPC compared to the PRIM samples (Figure 4B), but only in ADT and CRPC (Figure 4D, Supplementary Figure S1A,B), but MKI67 expression correlated with AR-Vs only in ADT and CRPC samples (Figure 4D, Supplementary Figure S1A,B). A similar upregulation of the PRIM MET, ADT and CRPC cohort was detected for the ubiquitin-conjugating enzyme E2C *(UBE2C)* in comparison to the CTRL, BPH and PRIM groups (Supplementary Figure S2A). The progressive loss of AR target gene expression, in combination with an increasing proliferative index, may hint at a progredient dedifferentiation of tumors ranging from the PRIM to CRPC cohorts. 

The expression of UBE2C is driven by AR-V- and not by AR-signaling in CRPC tumors [31]. Our analyses support this finding, as UBE2C expression was significantly upregulated only in CRPC tumors expressing AR-Vs (Figure 4E, Supplementary Figure S2B). In the same group, we found a significant association between AR-V expression with increased proliferation (MKI67) and the AR-V^+^-dependent elevation of AR target gene expression, while in AR-V negative CRPCs, AR target gene induction was in the range of the CTRL group (*p* = 0.003; Figure 4C). We did not find evidence for a correlation between AR pathway activity and *PTEN* or *RB1* expression in any of the groups (all *p* > 0.05). 

In both ADT and CRPC groups (Figure 4D), significantly higher *MKI67* mRNA expression was evident in tumors expressing AR-V splice variants. *PCNA* expression strongly correlated with the *MKI67* expression (Pearson’s r 0.52, *p* < 2.2 × 10^−16^; not shown) without any evidence of dependence on AR-Vs (Supplementary Figure S1C,D).

### 3.4. Homologous Recombination DNA Repair Activity Depends on the Presence of AR-Vs and AR Pathway Activation

**Figure 5:**
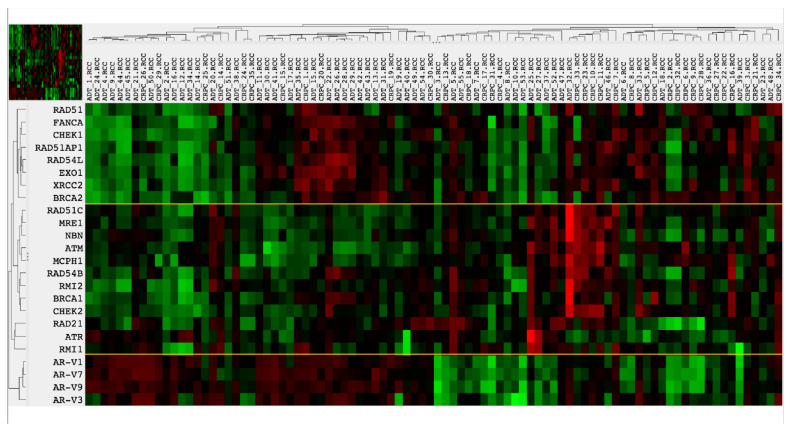
Heatmap plot of mRNA expression levels of DNA repair genes and AR-V splice variants as a quantitative parameter (**left side**). Samples represent pooled tumor samples from ADT and CRPC groups. Note similarities in the expression levels of the upper cluster of DNA repair genes (RAD51 to BRCA2) and AR-V splice variants, with the exception of 15 samples on the **left side** (mostly ADT samples). Clusters are separated by yellow lines.

Overall, the mRNA expression of 20 genes associated with HR DNA repair was analyzed in our study. Unsupervised heatmap clustering analysis showed evidence of two major clusters of DNA repair genes, one of them containing *BRCA1* and the other containing *BRCA2* expression. With the introduction of AR-Vs in this analysis as a quantitative parameter, AR-V expression was preferentially associated with the CRPC phenotype (11/45 in the AR-V low-expressing group vs. 23/45 in the AR-V high-expressers, Figure 5). In the AR-V high-expressing group, DNA repair gene expression was more abundantly deregulated as compared to the AR-V low-expressing group, where a more stable expression of DNA repair genes was observed.

**Figure 6:**
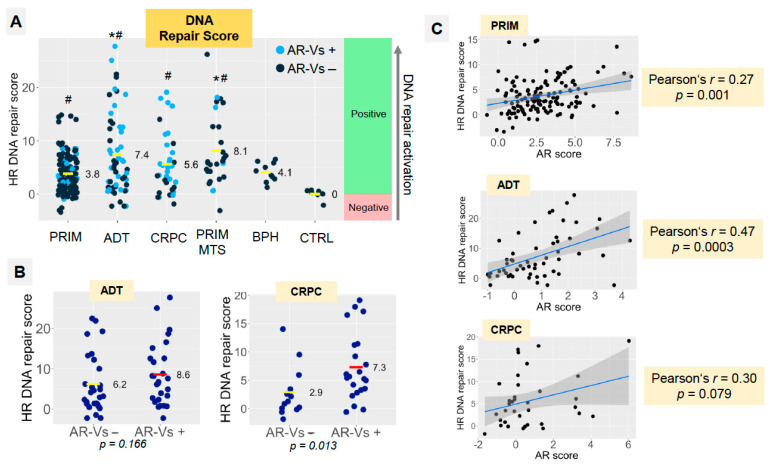
(**A**) Distribution of DNA repair scores in study groups (cumulative score based on mRNA expression of 20 DNA repair genes). Light blue points express any of the AR-V splice variants (V1, V3, V7, V9); dark blue points do not express AR-V splice variants. (**B**) Dependence of DNA repair score on the presence of AR-V splice variants (V1, V3, V7, V9) in ADT and CRPC groups. *p*-levels calculated using Mann–Whitney U-test. Statistical significance (*p* < 0.05): * vs. PRIM group, # vs. CTRL group. (**C**) Correlation analysis shows dependence of the AR score and DNA repair score in PRIM, ADT and CRPC groups.

To quantify DNA repair gene expression, we calculated a DNA repair activity score (DNA-R score) from the expression levels of 20 genes (excluding *PARP1* and *PARP2*) using CTRL samples as a reference, analogous to the AR score introduced above (Figure 6 and Figure S3). The DNA-R score significantly increased in all groups, including BPH, compared to the reference group (Figure 6A). In the presence of AR-Vs, the DNA-R score increased significantly in CRPCs. We observed a similar trend in the ADT group (Figure 6B). In the PRIM and ADT groups, as well as in the CRPC group (statistical trend), the DNA-R score significantly correlated with the AR score (Figure 6C). In linear regression models, the effect of proliferation on DNA repair was clearly visible (Table 2, lower panel). However when accounting for this effect, residual coefficient estimates revealed a positive association between the presence of AR-Vs and enhanced DNA repair in both primary tumors (ADT, *p* = 0.0377) and tumors that that underwent androgen deprivation therapy (ADT, *p* = 0.0297). Statistical analysis revealed a strong trend for CRPC within the latter group (Table 2, upper panel). An analysis of the CRPC group (*p* = 0.0551) showed a strong trend in the same direction in the respective regression models. The DNA-R score was not found to be correlated with *RB1*, *PTEN* or *ERG* mRNA expression (all *p* > 0.05).

**Figure 7:**
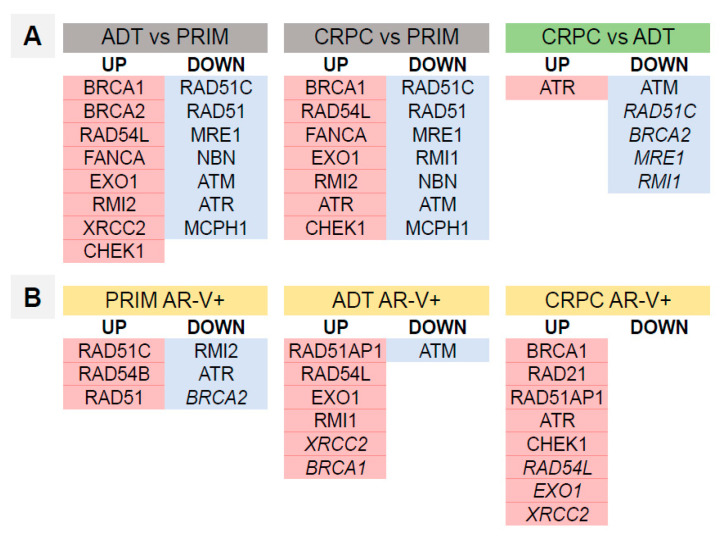
DNA repair genes differentially expressed in samples from different ADT, CRPC and PRIM groups (*p* < 0.05; in italic—*p* = 0.05–0.1) in relation to AR-V expression. Detailed expression analysis of individual DNA repair genes is provided in Supplementary Figures S4–S7. Upregulated genes are shown with a red background and downregulated genes with a blue background. (**A**) Cumulative analysis of all samples in study groups independent of AR-V status. (**B**) Analysis of genes affected in samples positive for any of AR-V splice variants (V1, V3, V7, V9) compared to those without AR-V splice variant expression.

An analysis of expression and dependence on the presence of AR-Vs for single HR DNA repair genes is presented in Supplementary Figures S4–S7. The trends in mRNA expression for the individual DNA repair genes were similar to the ADT and CRPC groups. Some genes (*ATM*, *RAD51C*, *BRCA2*, *MRE1*, *RMI1*) were more profoundly downregulated in the CRPC group (Figure 7A). A number of DNA repair genes showed statistically significant altered expression in the PRIM, ADT and CRPC groups in the presence of AR-Vs (Figure 7B).

It is known that DNA repair genes are also involved in various aspects of cell cycle progression. Gene ontology (GO) analyses of our gene set further validated the involvement of our gene set in DNA repair, as compared to the cell cycle and mitotic processes (Supplementary Figure S11A). We found BRCA1 genes as well as the BRCA2 cluster upregulated in processes related to DNA repair, but only to a low extent were they involved in cell cycle-related pathways. Ranking the pathways using GO Panther hierarchical cluster analysis (Supplementary Figure S11B) revealed our gene set to be significantly linked to multiple DNA repair pathways (DNA repair, double-strand break repair, double-strand break repair via homologous recombination, cellular response to DNA damage stimulus, recombinational repair, DNA recombination), followed by cell cycle pathways. It is striking that DDR pathways showed up to five times higher “fold enrichment” (13.91–62.22) compared to cell cycle pathways (9.25–13.34).

*PARP1* and *PARP2* mRNA expression was not found to correlate with the AR score in any of the groups. Both *PARP1* and *PARP2* were statistically significantly downregulated in the CRPC group compared to primary tumors (both *p* < 0.05; Supplementary Figure S8), where were not dependent on the presence of AR-Vs.

### 3.5. AR-V7 Enhances DNA Double-Strand Break Repair in an In Vitro PCA Model

**Figure 8:**
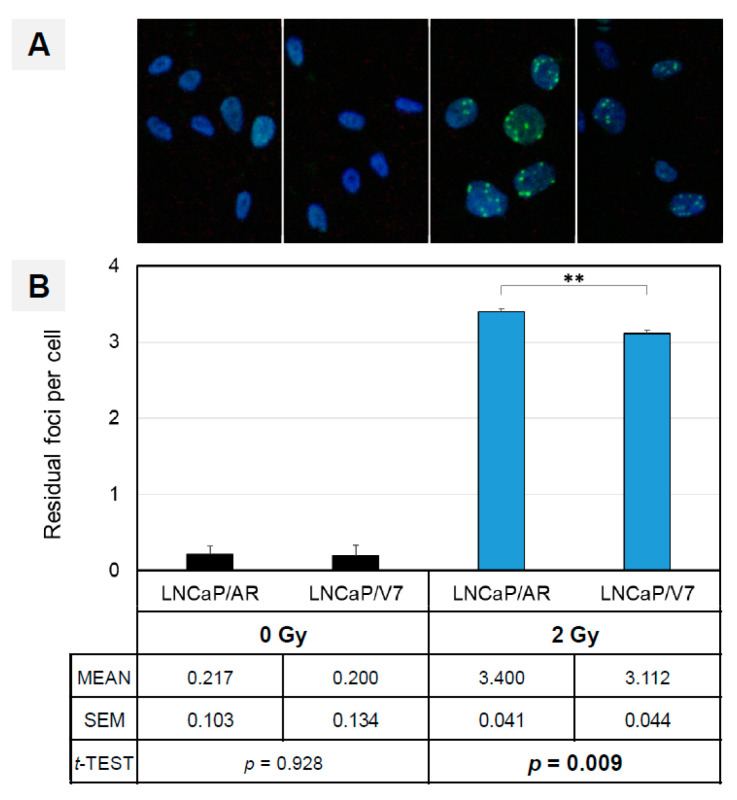
(**A**) The presence of AR-V7 enhances DNA repair in vitro. Increased DNA repair is visualized by a diminished number of residual γH2Ax foci 24 h after irradiation. Nuclear counterstain with DAPI from left to right: LNCaP/AR (0 Gy), LNCaP/V7 (0 Gy), LNCaP/AR (2 Gy), LNCaP/V7 (2 Gy). (**B**) Quantification of residual γH2A.X foci 24 h after irradiation (2 Gy, blue). Non-irradiated cells were used for comparison (0 Gy, black). In total, >200 nuclei were counted per cell line, irradiative condition and experiment, with a mean of three independent experiments. ** = *p* < 0.01.

The induction of DNA repair genes by AR splice variants can be measured by γH2A.X foci formation after the introduction of double-strand breaks. We used the LNCaP/V7 PCA in vitro tumor model for irradiation and assayed for γH2A.X foci formation over time. In contrast to primary foci, which correlate in number with DSBs, residual foci indicate the number of DBSs in the repair process [32]. We screened for foci formation at three timepoints (1 h, 24 h and 48 h after irradiation) and verified a strong induction of γH2AX focus formation at the early timepoint (1 h), a gradual decline of foci after 24 h and an almost complete loss of detectable foci 2 days after irradiation (Supplementary Figure S12). Compared to LNCaP cells overexpressing AR-FL (Supplementary Figure S10), we observed a modest but significantly reduced number of residual foci 24 h after irradiation (−8.5%, *p* < 0.01) in the presence of AR-V7, indicating accelerated DNA repair in LNCaP cells containing this AR splice variant [32] (Figure 8). Consequently, the presence of AR-V7 in PCA cells improves DNA repair provoked by X-ray irradiation.

### 3.6. In Vitro Validation of Findings in Clinical Samples

**Figure 9:**
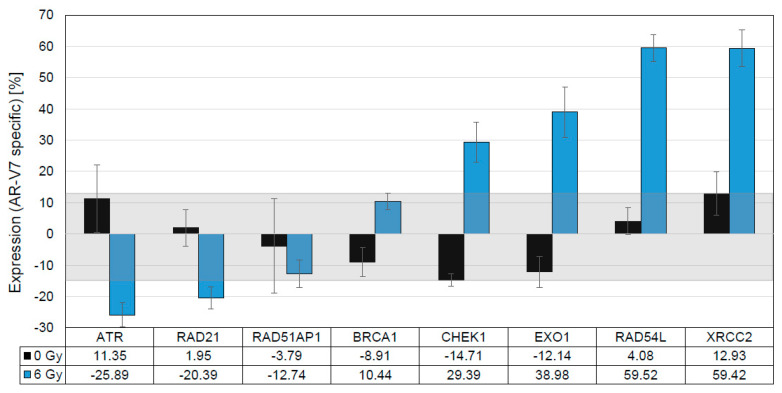
Validation of upregulated DNA repair genes of the AR splice variant expressing CRPC. LNCaP overexpressing AR-V7 served as a surrogate for CRPCs expressing AR-Vs. DNA repair genes upregulated in CRPC+AR-Vs (Figure 7B) were validated by qRT-PCR under irradiated (6 Gy, IR, blue bars) and non-irradiated (0 Gy, Ø, black bars) androgen-deprived conditions. The latter condition, with generally lower AR-V7-specific expression, served as the threshold (light gray box) to identify genes (CHEK1, EXO1, RAD54L, XRCC2) strongly upregulated by irradiation.

With the first proof that AR-V7 expression in tumor cells enhances DNA repair, our next step was to investigate the AR-V7-specific regulation of DNA repair gene expression (mRNA). DNA repair gene upregulation was observed in CRPC tumors (Figure 7B) expressing androgen receptor splice variants (CRPC AR-V+) in comparison to ADT refractory tumors in the absence of AR-Vs. We translated the CRPC AR-V+ phenotype in our tumor model to LNCaP cells expressing AR-V7, while androgen-resistant LNCaP cells overexpressing AR-FL mimicked the reference cohort (CRPC) [33]. We induced DNA damage in order to study the transcriptional regulation of DNA repair genes in vitro 6 h after irradiation. We observed only a minimal alteration of DNA repair genes in unirradiated reference samples of both LNCaP/AR and LNCaP/V7 cells. This finding disproves the assumption of a gross impact of AR-V7 on repair genes prior to DNA damage (Figure 9). Upon irradiation, however, a set of four genes (CHEK1, EXO1, RAD54L, XRCC2) was strongly upregulated, specifically in cells expressing AR-V7, confirming and validating the expression data generated from clinical samples, as described above. These similarities point to the representative nature of LNCaP/V7 cells as an in vitro model of CRPC tumor cells that express AR splice variants (CRPC AR-V+, Figure 7B).

**Figure 10:**
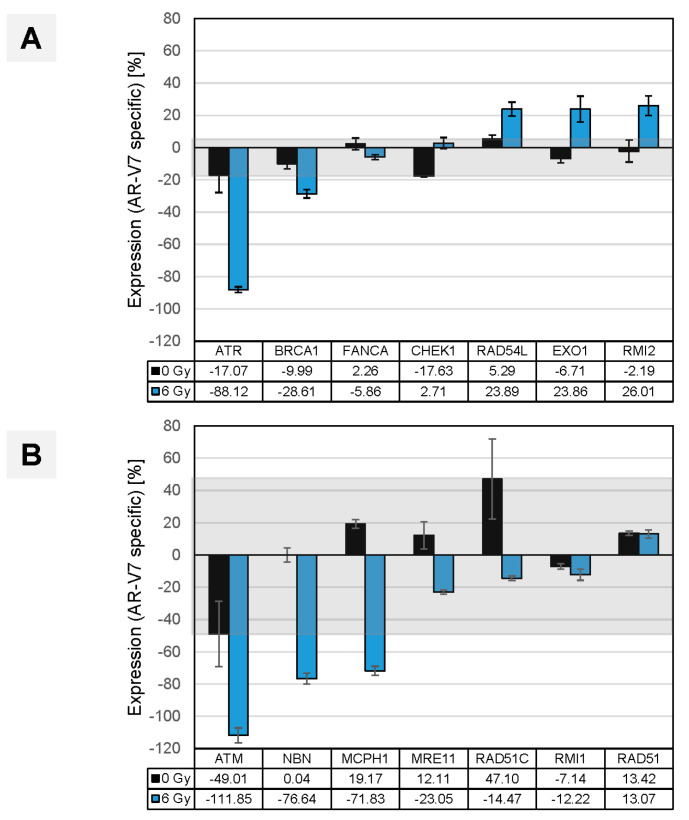
(**A**) Impact of AR splice variants in clinical CRPC via the validation of deregulated DNA repair genes in vitro. LNCaP cells overexpressing AR-V7 served as a surrogate for CRPCs expressing AR splice variants. DNA repair genes deregulated in CRPC vs. PRIM (Figure 7A) were validated by qRT-PCR under irradiated (6 Gy, IR, blue bars) and non-irradiated (0 Gy, Ø, black bars), androgen-deprived conditions. The latter condition, with generally lower AR-V7-specific expression, served as the threshold (light grey box) to identify genes visibly upregulated by irradiation such as RAD54L, EXO1, RMI2. (**B**) Under the same experimental conditions, genes such as ATM, NBN, MPCH1 were confirmed as downregulated.

A similar in vitro analysis was performed to corroborate the results achieved by comparing clinical CRPC and PRIM sub-cohorts (CRPC vs. PRIM; Figure 7A), where alterations in DNA repair gene expression were associated with the CRPC phenotype. *In vitro*, clinical CRPC was phenocopied by our LNCaP/V7 cell line, while the PRIM phenotype was represented by androgen-responsive LNCaP cells. As a primary result in the absence of DNA damage, we observed minor alterations in DNA repair gene mRNA expression. DNA repair induced by double-strand breaks resulted in a substantial shift in a subset of genes (RAD54L, EXO1, RMI2; Figure 10A), earlier identified as upregulated in clinical CRPC samples. Three additional genes (ATM, NBN, MCPH1) were confirmed as downregulated in vitro in the LNCaP/V7 CRPC model (Figure 10B). The genes identified by in vitro analyses can be equally assigned to either the group of DNA damage sensors (ATM, CHEK1/CHK1, MCPH1, NBN) or HR repair genes (EXO1, RAD54L, RMI2, XRCC2), in accordance with published data [3,5,8,34,35].

## 4. Discussion

The recognition of the commonly impaired DNA damage response by defects in HR has enabled new, targeted therapeutic interventions in many malignant tumors, including PCA [9,36]. The connection between the AR pathway and DNA repair in PCA proved to be so tight that, even in the absence of pathogenic mutations in DNA repair genes, there is a possibility of targeted therapeutic interventions using PARPi. ADT causes the significant downregulation of DNA repair genes given direct and indirect transcriptional regulation of the latter through AR [1,2,3,5,6,7,8,15,37]. This functional impairment of HR is sufficient to induce synthetic lethality under treatment with PARPi. Several major studies [5,6] provided a proof of principle for such synthetic lethality in cell line experiments, and two clinical trials have been conducted to date on patients with CRPC. A pilot study (NCI 9012) showed no differences in response rates between CRPC cohorts receiving abiraterone versus abiraterone/veliparib [38]. Abiraterone/olaparib, however, was effective in unselected patients with CRPC compared to abiraterone only [11], in particular, in a subgroup of patients with pathogenic mutations in HR DNA repair genes (NCT03732820) [39].

These studies [5,6] of synthetic lethality [11,38] analyzed AR function in the context of mutant DNA repair genes in CRPC tumors. AR splice variants showed activating effects in DNA repair genes similar to full-length androgen receptors [3,8], thus provoking the examination of the effect of AR-Vs on DNA repair genes in a clinical setting.

An appropriate in vitro model system has to fulfill several aspects to closely mimic the physiological situation of AR-V7-expressing PCA tumor cells [40]. The expression of AR-V7 is only observed in the context of full-length AR. The choice of an LNCaP cell model with high endogenous AR-FL expression caused by lentiviral transduction avoids clonal effects. To compensate for higher total AR expression in LNCaP/V7, we generated an LNCaP control cell line that overexpresses full-length AR (LNCaP/AR). Original LNCaP cells harbor the AR T878A mutation [41], which is known to cause aberrant AR behavior. However, the use of steroid-depleted media minimizes the effect of mutant AR signaling, and comparison with the control cell line LNCaP/AR, which overexpresses the wild-type full-length androgen receptor, ensures the analysis of AR-V7-specific effects in this in vitro tumor model. Hence, we are confident that our experimental setup allows for the analysis of AR-V7-specific effects in DDR even in the presence of the endogenous AR T878A mutation in LNCaP cells.

Cell lines such as VCaP might be used to analyze endogenous AR-V7 effects on DNA repair. However, VCaP cells are infected with and secrete the retrovirus Bxv-1, a xenotropic mouse leukemia virus, as shown by Sfanos et al. [42]. In the course of retroviral infection, γH2AX foci are detectable at sites of proviral integration [43], limiting the use of this assay to study DNA repair in VCaP cells.

Thus, we cannot exclude the impact of this virus on cellular physiology and AR-FL/AR-V7-signaling in these cells. We chose the transgenic approach using LNCaP cells, as these cells are not known to produce any viruses, closely mimicking the clinical situation. 

To the best of our knowledge, this is the first study to analyze the link between AR pathway activation, the presence of AR splice variants and the transcription of DNA repair genes in multiple subgroups of clinical PCA tumor samples that reflect the development of ADT resistance. While the upregulation of AR-V7 under ADT treatment in patient samples was already reported by Sharp et al. [21], our approach provides data for those subgroups, including a CRPC specimen. Additionally, these data were substantiated by cell line models, mimicking the CRPC phenotype of the clinical samples. Importantly, we provide evidence that crucial components of the DNA repair machinery might be induced in the presence of AR-Vs in CRPC. Thus, approximately two-thirds of patients with CRPC and half of the patients on ADT (but still hormone-sensitive) in our study harbored AR-V1, -V3, -V7, and -V9. Additionally, our study demonstrated the presence of AR-Vs is correlated to the increased activity of the AR pathway measured by the AR score in CRPC. Furthermore, the cellular proliferation index doubled in CRPC in the presence of AR-Vs as measured by MKI67 expression (Figure 4C,D).

This study shows that the expression of DNA repair genes, summarized as a DNA repair score, is 2.5 times higher in the presence of AR-Vs in CRPC (Figure 6B,C). This validates the results of previous experimental studies, which showed that, even in the absence of AR-FL, AR splice variants can provide the necessary transcriptional support for DNA repair genes [3] and the loss of AR-Vs sensitizes them to ionizing radiation [8]. As was demonstrated by Luo et al. [23], AR-V7 significantly promotes the DDR of PCA cells under severe DNA damage. The impact of AR-Vs on the DDR machinery may have clinical implications, as the activity of DNA repair in CRPC in the presence of AR-V splice variants seems to be similar or even higher than in hormone-naïve tumors. This may explain the rather inconclusive findings concerning the combination therapy of ADT with PARPi [11,38], implying that AR-Vs may be predictive of the efficacy of synthetic lethality-based therapeutic regimens (in a negative way), and this should be clarified in further studies.

The expression signature of DNA repair genes in primary tumors differs from that of tumors under ADT and CRPC (Figure 7). Most of the genes analyzed are similarly affected in ADT and CRPC samples: among upregulated genes (BRCA1, RAD54L, FANCA, EXO1, RMI2, XRCC1, CHEK1), the majority were found to have the same tendency in CRPC tumors in studies by Taylor et al. [44] and Grasso et al. [45] Only RAD51 was downregulated in our study, but it was upregulated in the aforementioned studies. Interestingly, the majority of regulated genes in our study (ADT and CRPC) were shown to be transcriptionally downregulated by androgen blockade in experimental studies [1,2,3,6,7,8,15,37], contradicting the findings of Taylor et al. [44], Grasso et al. [45] and our results. Our study provides evidence that AR-Vs may account for this discrepancy, as the genes induced by AR-Vs largely overlap with regulated genes in tumors under androgen depletion or CRPCs (compared to hormone-naïve tumors; Figure 7A,B).

Specifically, BRCA1, RAD54L, EXO1 and CHEK1 are identically upregulated. It is now tempting to draw the following conclusions. First, elevated levels of AR-Vs detectable during ADT provide transcriptional support for DNA repair gene activity. This is corroborated by two experimental studies utilizing PCA cell lines [3,8]. Second, the activating effect of AR-Vs applies to some, but not all, DNA repair genes. Indeed, AR-Vs and AR-FL transcriptomes are, to some extent, different [31], even in regard to DNA damage response genes [1,8,34,35]. However, the precise differences in the transcriptional effects of AR-FL and AR-Vs concerning DNA repair are, to date, understudied by far and warrant further investigations.

*PARP1* and *PARP2* were characterized earlier as important effectors of AR, functioning in positive regulatory loops [6,7,8,15]. In our study, mRNA expression in both genes was, to some extent, downregulated in CRPC samples compared to hormone-naïve tumors (Supplementary Figure S8) but did not show any dependency on AR splice variant expression. This could be related to the fact that mRNA expression in these genes is not a reliable measure of PARylation activity, which was also shown earlier [6].

Our analysis of clinical CRPC samples demonstrates the specific upregulation of DNA repair genes in two clinical settings: CRPC vs. PRIM and CRPC AR-V+ vs. CRPC AR-V-. This points to the initiation of DNA damage repair (DDR) in CRPC tumors. However, the precise mechanisms leading to AR-V-mediated modulations in DDR remain largely unknown. In our study, we constructed two in vitro cell line models simulating clinical CRPC tumor phenotypes with and without AR-V7 overexpression, derived from the initially hormone-sensitive LNCaP cell line (corresponding to the hormone-naïve phenotype in our experimental setup). In an in vitro CRPC model using LNCaP cells that overexpress AR-V7, DDR was induced by ionizing radiation. The presence of AR-V7 yielded a significant reduction in γH2A.X foci, which mark positions in the genome with actively ongoing DDR [32]. The resulting superior DDR has a beneficial impact on AR-V7 tumor cells. Upon DNA damage induced by irradiation, AR is known to translocate to the nucleus and initiate the expression of DNA repair genes such as XRCC2 [2]. AR splice variants such as AR-V7 are already located at a high fraction in the nuclei of primary PCA cells [46], while AR-FL remains cytoplasmatic in the absence of activating ligands. An augmented transcriptional activation of DNA repair genes in the presence of AR-Vs, therefore, appears plausible and was substantiated by our PCA tumor model.

DNA repair genes with de-regulated expression in CRPC clinical samples can either be classified in the group of DNA damage sensors (ATM, CHEK1/CHK1, MCPH1, NBN) or HR genes (EXO1, RAD54L, RMI2, XRCC2). Their altered expression was confirmed by our in vitro analyses. These data indicate that AR-Vs indeed play a multidirectional role in augmenting DDR in CRPC tumors (Figure 8, Figure 9 and Figure 10) and mirror the findings of Yin et al., 2017 [3], who reported a causal link between AR-Vs and DNA repair after irradiation.

Furthermore, we confirmed three genes (ATM, NBN and MCPH1) to be downregulated in clinical CRPC tumor samples in our in vitro models. Various publications reported a downregulation of genes with a potential tumor-suppressive function in DNA repair [40,47], with ATM being one of the crucial and most thoroughly studied. One publication showed an association between NBN mutations and high-grade PCA in a Polish patient cohort [48], suggesting a tumor-suppressive function in this gene in normal prostate tissue. MCPH1, also downregulated in our clinical CRPC tumor samples, was previously reported to be downregulated in prostate carcinoma [49]. MCPH1 is functionally tightly associated with ATM and NBN. MCPH1 recruits ATM and NBN to DNA damage repair foci as part of the early DNA damage response [50]. The functional loss of ATM or NBN, two genes that are downregulated in our study, is associated with poor survival in PCa patients [50,51,52] and sensitizes PARP inhibitor therapies. Androgen receptor splice variant expression, in contrast, appears to reactivate DNA damage repair gene expression previously thwarted by ADT. In summary, our experiments using the AR-V7 in vitro PCA model suggest that the PARPi sensitization of CRPC tumors will likely not occur in the presence of AR-Vs [8], despite the observed loss of ATM expression. To the best of our knowledge, for the first time, the AR-V-dependent alteration of DDR gene expression has been shown in CRPC patients (Figure 7), and accelerated DDR in an AR-V7-dependent CRPC tumor model was confirmed.

In contrast to our expectations, we found an independent impact of AR-V expression on DDR gene expression in the clinical cohort. The employment of a linear regression model provided not only a significant positive association of AR-V expression with proliferation but also independently for DNA repair gene expression in primary tumors (Table 2). Calculations for the other groups resulted in corresponding associations, concluding that AR-Vs have an independent impact on DDR gene expression. Corroborating these findings in vitro, analogous alterations of DDR gene expression, as found in the clinical samples, appeared within a timeframe that excludes the involvement of proliferative aspects of the functionality of AR-V7.

This study is not devoid of limitations, as DNA repair is a very complex process. Other important components of the DNA repair system, such as Ku70 protein [53], DNA protein kinase catalytic subunit [54] and some other genes, which have been shown to be AR-dependent, were not studied. Several other AR rearrangements (gene amplification and mutations) were also not targets of our study.

## 5. Conclusions

This study confirms the tight interconnection between AR signaling, alterations in AR expression and the transcription of DNA repair genes in clinical tumor samples and in vitro prostate cancer models. Of particular importance is the modulation of DNA repair gene expression in the presence of AR splice variants in CRPC. The expression of AR splice variants might be a reason for the reduced or absent effect of therapeutic concepts exploiting the principle of synthetic lethality between ADT and PARP inhibition. Thus, AR-Vs show potential as predictive biomarkers for the efficacy of PARPi therapy, as previously suggested [23].

## Figures and Tables

**Table 1:** Clinical characteristics of the study cohort.

	Number of Patients	Number of Samples
PCA, primary tumor, hormone-naïve	77	136
***pT-stage***		
pT1b (TURP)	20	
pT2	14	
pT3a	9	
pT3b	21	
pT4	3	
unknown	10	
***pN stage***		
pN0	32	
pN1	12	
pNx	33	
***Prostatectomy*** *ISUP/WHO grade group*		
ISUP 1	3	
ISUP 2	6	
ISUP 3	1	
ISUP 4	37	
ISUP 5	30	
***Morphology***		
Acinar adenocarcinoma	52	
Ductal/mixed adenocarcinoma ^§^	25	
PCA, metastases, hormone-naïve	23	28
PCA ADT *	42	55
PCA CRPC *^,#^	32	35
BPH	10	10
Benign prostate tissue without hyperplasia	7	7
Neuroendocrine carcinoma of the prostate	1	1
Sarcomatoid carcinoma ^$^	1	1
OVERALL	184	273

Comments: *—CRPC samples from primary (untreated) tumor in the prostate or from metastases; ^§^—only samples containing ductal adenocarcinoma were included in the analysis in case of mixed ductal/acinar morphology; ^#^—four samples of CRPC bone metastases that failed quality control are not showed here. ^$^—primary tumor sample, after androgen deprivation therapy.

**Table 2:** Estimates of associations between AR splice variants, proliferation and DNA repair, as obtained from fitting group-wise linear regression models with the DNA-R score as a dependent variable and splice variant and proliferation scores as independent variables. * *p* < 0.05, *** *p* < 0.0005.

**AR splice variants vs. DNA repair.**				
	**Patients**	**Coefficient Estimate**	**SD**	***p*-Value**
ADT	88	0.008013	0.003624	0.0297 *
ADT nonCRPC	53	0.007468	0.004472	0.101
CRPC	35	0.012147	0.006103	0.0551
Prim	136	0.080467	0.038329	0.0377 *
**AR splice variants vs. proliferation.**				
	**Patients**	**Coefficient Estimate**	**SD**	***p*-Value**
ADT	88	0.028671	0.002070	<2 × 10^−16^ ***
ADT nonCRPC	53	0.030869	0.002317	<2 × 10^−16^ ***
CRPC	35	0.023398	0.003974	1.5 × 10^−6^ ***
Prim	136	0.030435	0.002304	<2 × 10^−16^ ***

## Data Availability

Data generated in this study are available upon request.

## Supplementary Materials

The following supporting information can be downloaded at: https://www.mdpi.com/article/10.3390/cancers14184441/s1. Table S1: CodeSet gene panel used in the study with probe sequences for RNA expression analysis. Table S2: Primer sequences used for qPCR analysis. Table S3: Validation of DNA repair genes by qRT-PCR analysis that were significantly deregulated in clinical samples (CRPC + AR-V). Table S4: Validation of DNA repair genes by qRT-PCR analysis that were significantly deregulated in clinical samples (CRPC vs PRIM). Figure S1: MKI67 and PCNA mRNA expression in study groups. Figure S2: UBE2C mRNA expression in study groups. Figure S3: mRNA expression of 20 DNA repair genes in study groups and correlation of DNA repair and AR scores. Figure S4: mRNA expression of 20 DNA repair genes in dependence on AR-V status in the PRIM group. Figure S5: mRNA expression of 20 DNA repair genes in dependence on AR-V status in the CRPC group. Figure S6: mRNA expression of 20 DNA repair genes in dependence on AR-V status in the ADT group. Figure S7: mRNA expression of 20 DNA repair genes in dependence on AR-V status. Figure S8: mRNA expression of PARP1 (A) and PARP2 (B) in study groups. Figure S9: Separation of the impact of AR splice variants on proliferation and DNA repair using a linear regression model. Figure S10: Expression of total androgen receptor and splice variant 7 in the LNCaP/AR in vitro tumor models. Figure S11: Gene ontology analysis of genes de-regulated in clinical samples reveals association with DNA repair. Figure S12: AR-V7 improves DNA repair after irradiation in an in vitro tumor model.
