# Supplementary material for: Androgen Receptor Splice Variants Contribute to the Upregulation of DNA Repair in Prostate Cancer
Source: Cancers (Basel). 2022 Sep 13;14(18):4441. doi: 10.3390/cancers14184441 (PMC9496991; doi:10.3390/cancers14184441)
Supplement: Supplementary file 1 [file cancers-14-04441-s001.zip › cancers-1846664-supplementary.pdf]

Table S1: CodeSet gene panel used in the study with probe sequences for RNA expression analysis. Genes involved in this analysis are listed below with symbols, position of the Nanostring probe within the mRNA sequence and probe sequence. NCBI accession numbers are used for genes, ensemble transcript identifiers are used for AR isoforms. Housekeeping genes are marked in green, genes involved in DNA repair are in blue. All other genes, including AR-V genes, are labeled in black.

Gene symbol	Accession #	Position	Sequence
ABCF2	NM_007189.1	1541-1640	TCTCACCTTTGGAGTACATGATGAAGTGCTACCCAGAGATCAAGGAGAAGGAAGAAATGAGGAAGATCATTGGGCGATACGGTCTCACTGGGAAACAACA
AGR2	NM_006408.3	581-680	GACCCATCTCTGACAGTTAGAGCCGATATCACTGGAAGATATTCAAATCGTCTCTATGCTTACGAACCTGCAGATACAGCTCTGTTGCTTGACAACATGA
ALAS1	NM_000688.4	396-495	AGAAAGCAGGCAAATCTCTGTTGTTCTATGCCCAAAACTGCCCCAAGATGATGGAAGTTGGGGCCAAGCCAGCCCCTCGGGCATTGTCCACTGCAGCAGT
AR-V9	AR_V9.1	30-129	AAATGTTATGAAGCAGGGATGACTCTGGGAGGACAACTTACCTGAGCAAGCTGCTTTTTGGAGACATTTGCACATCTTTTGGGATCACGTT
AR-V3	ENST00000514029.1	2050-2149	CTCACATGTGGAAGCTGCAAGGTCTTCTTCAAAAGAGCCGCTGAAGGATTTTTCAGAATGAACAAATTAAAAGAATCATAATCAGACACTAACCCCAAGC
AR-V7	ENST00000514029.	12446-2545	GTCCATCTTGTCGTCTTCGGAAATGTTATGAAGCAGGGATGACTCTGGGAGAAAAATTCCGGGTTGGCAATTGCAAGCATCTCAAAATGACCAGACCCTG
AR-FL	NM_000044.4	3081-3180	TGCTCAAGACGCTTCTACCAGCTCACCAAGCTCCTGGACTCCGTGCAGCCTATTGCGAGAGAGCTGCATCAGTTCACTTTTGACCTGCTAATCAAGTCAC
AR45	NM_001011645.3	2278-2377	TGCTCCTGACATTGCCTGTCACTTTTTCCCATGATACTCTGGCTTCACAGTTTGGAGACTGCCAGGGACCATGTTTTGCCCATTGACTATTACTTTCCAC
AR-V1	NM_001348063.1	2368-2467	GTCGTCTTCGGAAATGTTATGAAGCAGGGATGACTCTGGGAGCAGCTGTTGTTGTTTCTGAAAGAATCTTGAGGGTGTTTGGAGTCTCAGAATGGCTTCC
ARv567es	GU208210.1	2139-2238	ACTGGGAGAGAGACAGCTTGTACACGTGGTCAAGTGGGCCAAGGCCTTGCCTGATTGCGAGAGAGCTGCATCAGTTCACTTTTGACCTGCTAATCAAGTC
ARF1	NM_001024227.1	1371-1470	CAATTCTGCATGGTCACAGTAGAGATCCCCGCAACTCGCTTGTCCTTGGGTCACCCTGCATTCCATAGCCATGTGCTTGTCCCTGTGCTCCCACGGTTCC
ATM	NM_138292.3	1324-1423	TGAAGATAAAGAACTTCAGTGGACCTTCATAATGCTGACCTACCTGAATAACACACTGGTAGAAGATTGTGTCAAAGTTCGATCAGCAGCTGTTACCTGT
ATR	NM_001184.2	566-665	AAGACTTGGTTTACCTCCATAGAAGAAATGTGATGGGTCATGCTGTGGAATGGCCAGTGGTCATGAGCCGATTTTTAAGTCAATTAGATGAACACATGGG
BRCA1	NM_007305.2	1276-1375	CATTAGATGATAGGTGGTACATGCACAGTTGCTCTGGGAGTCTTCAGAATAGAAACTACCCATCTCAAGAGGAGCTCATTAAGGTTGTTGATGTGGAGGA
BRCA2	NM_000059.3	116-215	GGGGACAGATTTGTGACCGGCGCGGTTTTTGTCAGCTTACTCCGGCCAAAAAAGAACTGCACCTCTGGAGCGGACTTATTTACCAAGCATTGGAGGAATA
CHEK1	NM_001114121.1	2226-2325	AGGGTGATGGATTGGAGTTCAAGAGACACTTCCTGAAGATTAAAGGGAAGCTGATTGATATTGTGAGCAGCCAGAAGATTTGGCTTCCTGCCACATGATC
CHEK2	NM_001005735.1	895-994	GGAGAGGTAAAGCTGGCTTTCGAGAGGAAAACATGTAAGAAAGTAGCCATAAAGATCATCAGCAAAAGGAAGTTTGCTATTGGTTCAGCAAGAGAGGCAG
DMC1	NM_007068.2	901-1000	GCAGCAAAAATTGGCCCAGATGTTGTCACGACTCCAAAAAATCTCAGAAGAATATAACGTGGCTGTTTTTGTGACCAATCAAATGACTGCCGATCCAGGA
ECI2	NM_006117.2	941-1040	AGTTAACAGCGGGAGAGGCATGTGCTCAAGGACTTGTTACTGAAGTTTTCCCTGATAGCACTTTTCAGAAAGAAGTCTGGACCAGGCTGAAGGCATTTGC
ERG	NM_001136155.1	342-441	GCGGTGAAAGAATATGGCCTTCCAGACGTCAACATCTTGTTATTCCAGAACATCGATGGGAAGGAACTGTGCAAGATGACCAAGGACGACTTCCAGAGGC
EXO1	NM_003686.3	2716-2815	GCCAGAGCCAGTGGGCTGAGCAAGAAGCCGGCAAGCATCCAGAAGAGAAAGCATCATAATGCCGAGAACAAGCCGGGGTTACAGATCAAACTCAATGAGC
FANCA	NM_000135.2	799-898	CTGAGAAGAACTGTGGAGCCTGAAAAAATGCCGCAGGTCACGGTTGATGTACTGCAGAGAATGCTGATTTTTGCACTTGACGCTTTGGCTGCTGGAGTAC
FASN	NM_004104.4	5388-5487	GAGGTGCTTGGCTACGCACGGTCGCTTCCTGGAAATTGGCAAATTCGACCTTTCTCAGAACCACCCGCTCGGCATGGCTATCTTCCTGAAGAACGTGACA
GDF15	NM_004864.2	181-280	ACTCCAGATTCCGAGAGTTGCGGAAACGCTACGAGGACCTGCTAACCAGGCTGCGGGCCAACCAGAGCTGGGAAGATTCGAACACCGACCTCGTCCCGGC
GFM1	NM_024996.5	1611-1710	GAAATCTATGCTCAGAGGCTGGAAAGAGAGTATGGCTGTCCTTGTATCACAGGAAAGCCAAAAGTTGCCTTTCGAGAGACCATTACTGCCCCTGTCCCGT
HPRT1	NM_000194.1	241-340	TGTGATGAAGGAGATGGGAGGCCATCACATTGTAGCCCTCTGTGTGCTCAAGGGGGGCTATAAATTCTTTGCTGACCTGCTGGATTACATCAAAGCACTG
KLK3	NM_001030049.1	434-533	GTGTGTGGACCTCCATGTTATTTCCAATGACGTGTGTGCGCAAGTTCACCCTCAGAAGGTGACCAAGTTCATGCTGTGTGCTGGACGCTGGACAGGGGGC
MCPH1/ BRIT1	NM_024596.2	643-742	CCACCTCTTCCCAAATGATTCAGCAGTCTCATGATAATCCAAGTAACTCTCTGTGTGAAGCACCTTTGAACATTTCACGTGATACTTTGTGTTCAGATGA
MKI67	NM_002417.2	4021-4120	AGCAGATGTAGAGGGAGAACTCTTAGCGTGCAGGAATCTAATGCCATCAGCAGGCAAAGCCATGCACACGCCTAAACCATCAGTAGGTGAAGAGAAAGAC
MRE1	NM_005591.3	506-605	TTCCATGGGTGAACTATCAAGATGGCAACCTCAACATTTCAATTCCAGTGTTTAGTATTCATGGCAATCATGACGATCCCACAGGGGCAGATGCACTTTG
NBN	NM_002485.4	1061-1160	GACTACAAAGAATTACTGTGATCCTCAGGGCCATCCCAGTACAGGATTAAAGACAACAACTCCAGGACCAAGCCTTTCACAAGGCGTGTCAGTTGATGAA
PARP1	NM_001618.3	3017-3116	AAGGTTTGGGCAAAACTACCCCTGATCCTTCAGCTAACATTAGTCTGGATGGTGTAGACGTTCCTCTTGGGACCGGGATTTCATCTGGTGTGAATGACAC
PARP2	NM_005484.3	1155-1254	GTTATGAGTTCAAAGTGATTTCCCAGTACCTACAATCTACCCATGCTCCCACACACAGCGACTATACCATGACCTTGCTGGATTTGTTTGAAGTGGAGAA
PCNA	NM_002592.2	281-380	GGTGTTGGAGGCACTCAAGGACCTCATCAACGAGGCCTGCTGGGATATTAGCTCCAGCGGTGTAAACCTGCAGAGCATGGACTCGTCCCACGTCTCTTTG
PGK1	NM_000291.2	1031-1130	GCAAGAAGTATGCTGAGGCTGTCACTCGGGCTAAGCAGATTGTGTGGAATGGTCCTGTGGGGGTATTTGAATGGGAAGCTTTTGCCCGGGGAACCAAAGC
PTEN	NM_000314.6	5345-5444	CTTCAGATACTCTTGTGCTGTGCAGCAGTGGCTCTGTGTGTAAATGCTATGCACTGAGGATACACAAAAATACCAATATGATGTGTACAGGATAATGCCT
RAD21	NM_006265.2	1081-1180	GATGAGGATGATAATGTATCAATGGGTGGGCCTGATAGTCCTGATTCAGTGGATCCCGTTGAACCAATGCCAACCATGACTGATCAAACAACACTTGTTC
RAD51	NM_133487.2	567-666	AGACCACCAGACCCAGCTCCTTTATCAAGCATCAGCCATGATGGTAGAATCTAGGTATGCACTGCTTATTGTAGACAGTGCCACCGCCCTTTACAGAACA
RAD51AP1	NM_001130862.1	1126-1225	TCTGTGAAGTCTCCCAATCAGAGTCTCCGCCTTGGCTTGTCCAGATTAGCACGAGTTAAACCTTTGCATCCAAATGCCACTAGCACCTGAGTGTGGTACA
RAD51C	NM_002876.2	301-400	GCACTGGAACTTCTTGAGCAGGAGCATACCCAGGGCTTCATAATCACCTTCTGTTCAGCACTAGATGATATTCTTGGGGGTGGAGTGCCCTTAATGAAAA
RAD54B	NM_012415.2	827-926	TAGACAGAATGATTTCCAAAATTGCAAACCACGCCATGACCCATATACGCCAAATTCCCTCGTTATGCCACGACCAGATAAGAATCACCAGTGGGTATTC
RAD54L	NM_003579.2	1436-1535	ATGAACCAGCGTGGAGCCAGGGTGTCTTCTCCCATCCTCATCATTTCCTATGAGACCTTCCGCCTTCATGTTGGAGTCCTCCAGAAAGGAAGTGTTGGTC
RB1	NM_000321.1	2111-2210	CCTATCTCCGGCTAAATACACTTTGTGAACGCCTTCTGTCTGAGCACCCAGAATTAGAACATATCATCTGGACCCTTTTCCAGCACACCCTGCAGAATGA
RMI1	NM_024945.2	528-627	TGTTAACTTGAGTCAGGCCCAAATGAATAAACAAGTGTTTGAGCAGTGGCTCCTTACTGATCTGAGGGATTTGGAGCATCCTCTTTTACCCGATGGCATT
RMI2	NM_152308.1	891-990	AAGACAGACTGTGTAAAAAAGGAATGACATCCTGGCTCCTCATCTTCTTCATCAGCAACTACCATAACCAGTTTGCGAGTCAAATGGCATTTCCTAACGG
SEC61A1	NM_013336.3	2246-2345	TCTGTGCACCTATTGGCTCTTCTAGCTGACTCTTCTGGTTGGGCTTAGAGTCTGCCTGTTTCTGCTAGCTCCGTGTTTAGTCCACTTGGGTCATCAGCTC
STIL	NM_001048166.1	2477-2576	GGAAGCACAGTCTTCCCCTGGCTTGCACATGAGAAAAGGTGTAAGCATTGCTGTGAGCACAGGTGCTAGCTTGTTTTGGAATGCAGCAGGTGAGGATCAA
TM4SF1	NM_014220.2	96-195	AATCGCAGTATTTAAGAGGTAGCAGGAATGGGCTGAGAGTGGTGTTTGCTTTCTCCACCAGAAGGGCACACTTTCATCTAATTTGGGGTATCACTGAGCT
TMPRSS2	NM_005656.3	1080-1179	AGACAATCTTTCATGTTCTATGGAGCCGGATACCAAGTAGAAAAAGTGATTTCTCATCCAAATTATGACTCCAAGACCAAGAACAATGACATTGCGCTGA
UBE2C	NM_181803.1	270-369	CTTTTAAGAAGTACCTGCAAGAAACCTACTCAAAGCAGGTCACCAGCCAGGAGCCCTGACCCAGGCTGCCCAGCCTGTCCTTGTGTCGTCTTTTTAATTT
XRCC2	NM_005431.1	537-636	CTGTCAGCTTTTTACTGGATAGACCGCGTCAATGGAGGAGAAAGTGTGAACTTACAGGAGTCTACTCTGAGGAAATGTTCTCAGTGCTTAGAGAAGCTTG

Table S2: Primer sequences used for qPCR analysis

Gene	FW-Primer	RV-Primer
ATM	TGGTGCTATTTACGGAGCTGA	AGCCTGAAGTACACAGAGAACA
ATR	AGGCCAAAGGCAGTTGTATTG	CAAATGACAGGAGGGAGTTGCT
BRCA1	TGCGGGAGGAAAATGGGTAG	CTGGGATTCTCTTGCTCGCT
BRCA2	GACTCTGCCGCTGTACCAAT	GTGGACAGGAAACATCATCTGC
CHEK1	CATGGCAGGGGTGGTTTATCT	CGAAATACTGTTGCCAAGCCA
EXO1	TGCGTGGGATTGGATTAGCA	TGGCCCGAATAAACCCGTTG
FANCA	GGACCTGAATGCCCTTTTGC	AGGCTTGATCCTGCAAAGCA
MCPH1/ BRIT1	AAATCTTTCCCCCACCTCTTCC	ATGAGTGTAAGCCACCAGCA
MRE11	TCCCAGAGGAGCTTGACTGA	CCTCTGACTGCATCTTTCTCCA
NBN	GCAGAAATTGGATTGGCGGT	AAGGCTTGGTCCTGGAGTTG
RAD21	CAAAGCCCATGTGTTCGAGTG	GTCCTGATGTCCGTAATGCC
RAD51	TCAACACAGACCACCAGACC	CTGAAAGCTCACCTCGACCC
RAD51AP1	TGCGGCCTGTGAGACATAAG	TCCTTTGGTGCTGTTCTGGA
RAD51C	AAACCCTCCGAGCTTAGCAA	TGTGACTCAGATGTACCAGCA
RAD54B	ACTGTTTCCCTCTTGTGGATGT	AGCTCCACATCTGCCATTCA
RAD54L	GCTGGCCAAGAGAAAACCTG	ATCTGGGTCTCACTGCTGGA
RMI1	TGTCATCAAATCCTGTGCTGCT	TGCAGCTAAAAGCCAAGTTTCA
RMI2	CGGCCCTGTCTAGTCCCA	CCTCCAGTTCCCACATACTTTC
XRCC2	GGGCGATGTGTAGTGCCTT	CTTCTACCTTCAAGTCGGGCA
PPIA	GCTGGACCCAACACAAATGG	GGCCTCCACAATATTCATGCCT

Table S3: Validation of DNA repair genes by qRT-PCR analysis that were significantly deregulated in clinical samples. Comparison of androgen-resistant AR-V7 overexpressing cells (LNCaP/V7) with androgen-resistant AR-FL overexpressing cells (LNCaP/AR) mimics the results of the clinical data obtained for CRPC AR-V+ (**Fig. 6B**). DNA repair was provoked by irradiation and AR-V7 specific gene regulation (DCC IR) was analyzed by qRT-PCR using the $\Delta\Delta C_{\tau}$ method. Non-irradiated cells (DCC) were checked in parallel. Genes significantly upregulated in the CRPC AR-V+ analysis were marked.

Gene	DCC	DCC IR	CRPC + AR-V
ATM	28%	-57%	
ATR	11%	-26%	UP
BRCA1	-9%	10%	UP
BRCA2	-2%	26%	
CHEK1	-15%	29%	UP
EXO1	-12%	39%	UP
FANCA	20%	43%	
MCPH1	-12%	-58%	
MRE11	9%	-30%	
NBN	-13%	-49%	
RAD21	2%	-20%	UP
RAD51	-23%	28%	
RAD51AP1	-4%	-13%	UP
RAD51C	15%	-3%	
RAD54B	9%	23%	
RAD54L	4%	60%	UP
RMI1	2%	16%	
RMI2	0%	50%	
XRCC2	13%	59%	UP

Table S4: Validation of DNA repair genes by qRT-PCR analysis that were significantly deregulated in clinical samples. Comparison of androgen-resistant AR-V7 overexpressing cells (LNCaP/V7) with androgen-sensitive LNCaP wt cells mimics the results of the clinical data obtained for CRPC vs PRIM (**Fig. 6A**). DNA repair was provoked by irradiation and the impact of the AR-V7 splice variant on the CRPC phenotype (DCC IR) was analyzed by qRT-PCR using the $\Delta\Delta C_\tau$ method. Non-irradiated cells (DCC) were checked in parallel. Genes significantly deregulated in the CRPC vs PRIM analysis were marked.

Gene	DCC	DCC IR	CRPC vs PRIM
ATM	-49%	-112%	DOWN
ATR	-17%	-88%	UP
BRCA1	-10%	-29%	UP
BRCA2	-2%	-24%	
CHEK1	-18%	3%	UP
EXO1	-7%	24%	UP
FANCA	2%	-6%	UP
MCPH1	19%	-72%	DOWN
MRE11	12%	-23%	DOWN
NBN	0%	-77%	DOWN
RAD21	4%	-25%	
RAD51	13%	13%	DOWN
RAD51AP1	39%	7%	
RAD51C	47%	-14%	DOWN
RAD54B	-9%	-7%	
RAD54L	5%	24%	UP
RMI1	-7%	-12%	DOWN
RMI2	-2%	26%	UP
XRCC2	3%	23%	

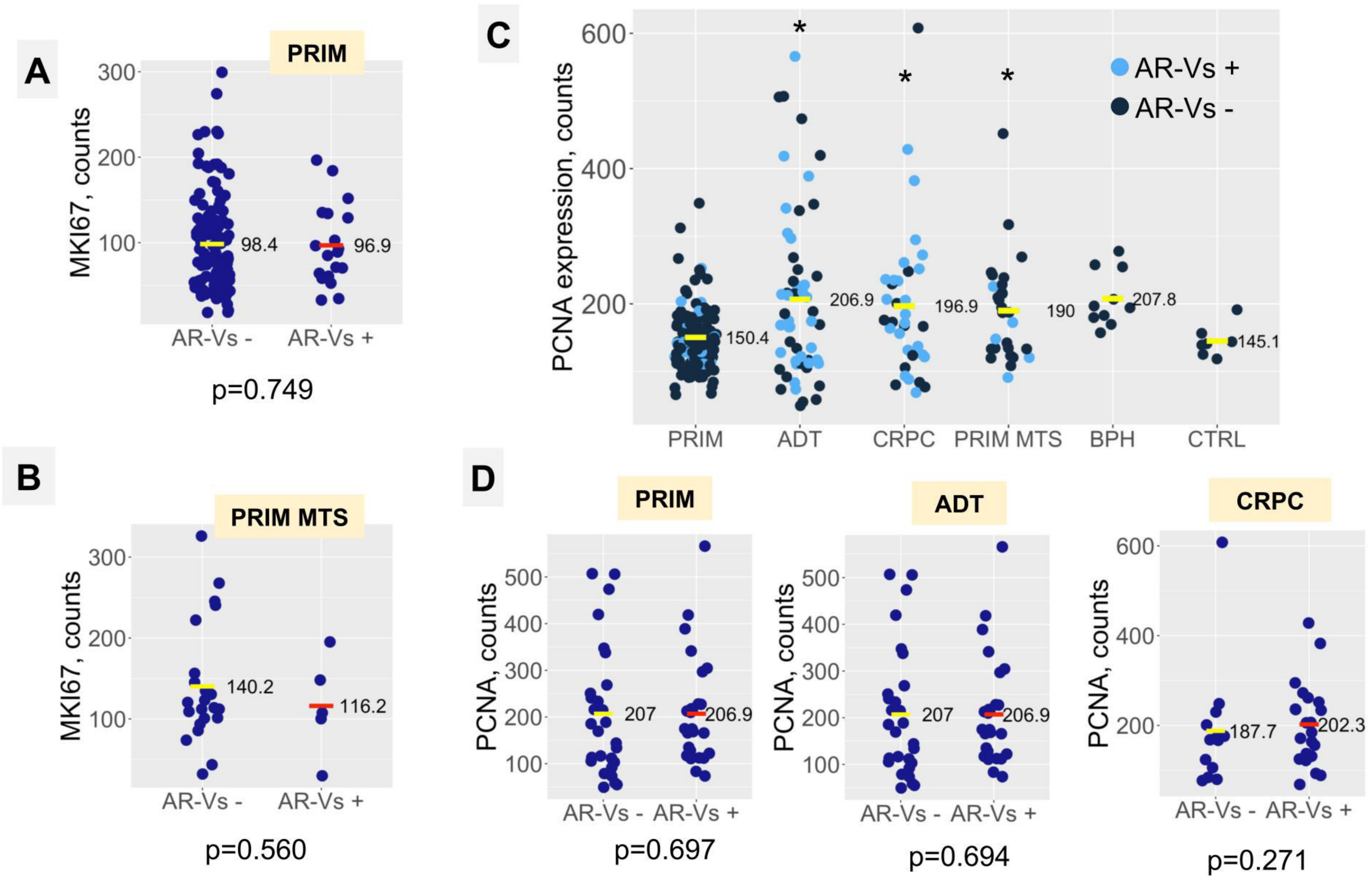

Figure S1: MKI67 and PCNA mRNA expression in study groups. Light blue points express any of AR-V splice variants (V1, V3, V7, V9), dark blue points do not express AR-V splice variants. *p*-levels were calculated using the Mann-Whitney U-test. * vs PRIM

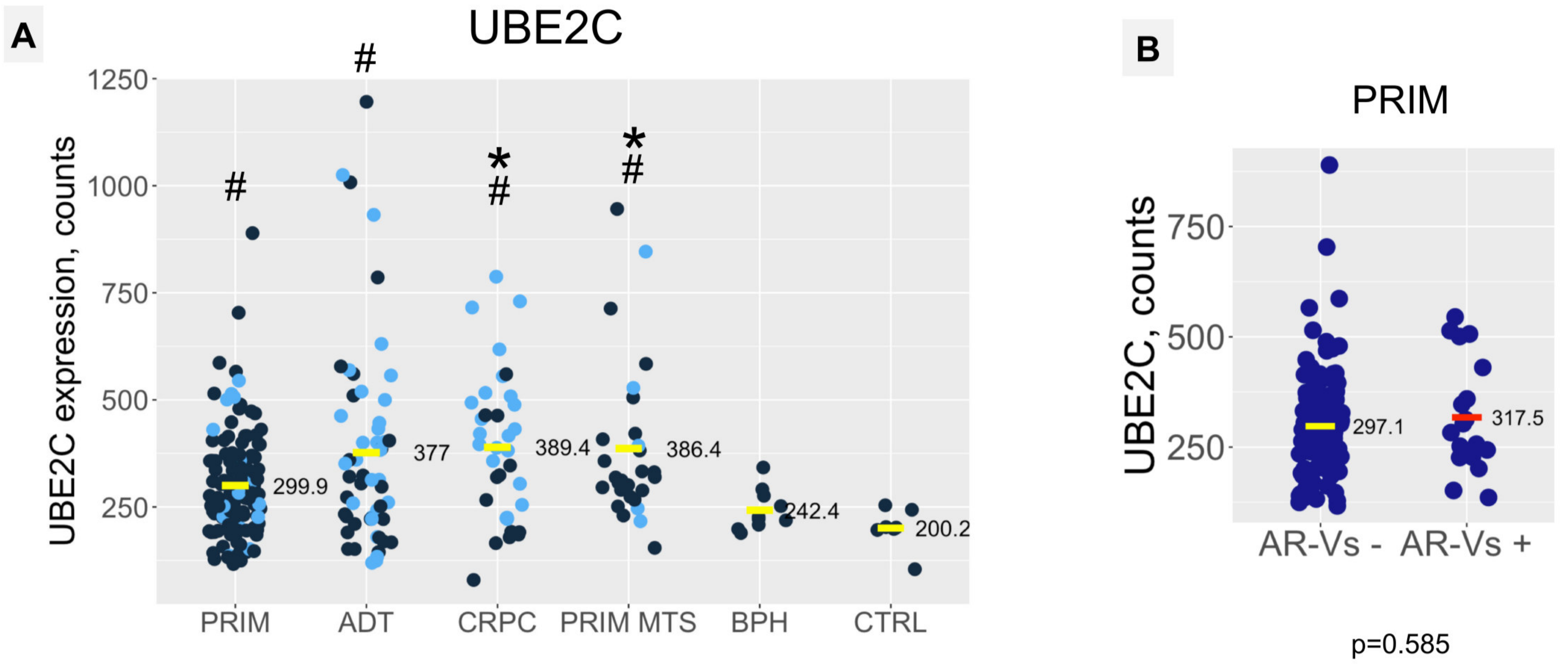

Figure S2: UBE2C mRNA expression in study groups. Light blue points express any of AR-V splice variants (V1, V3, V7, V9), dark blue points do not express AR-V splice variants.p-levels were calculated using the Mann-Whitney U-test. Statistical significance ($p < 0.05$): * vs PRIM group, # vs CTRL group.

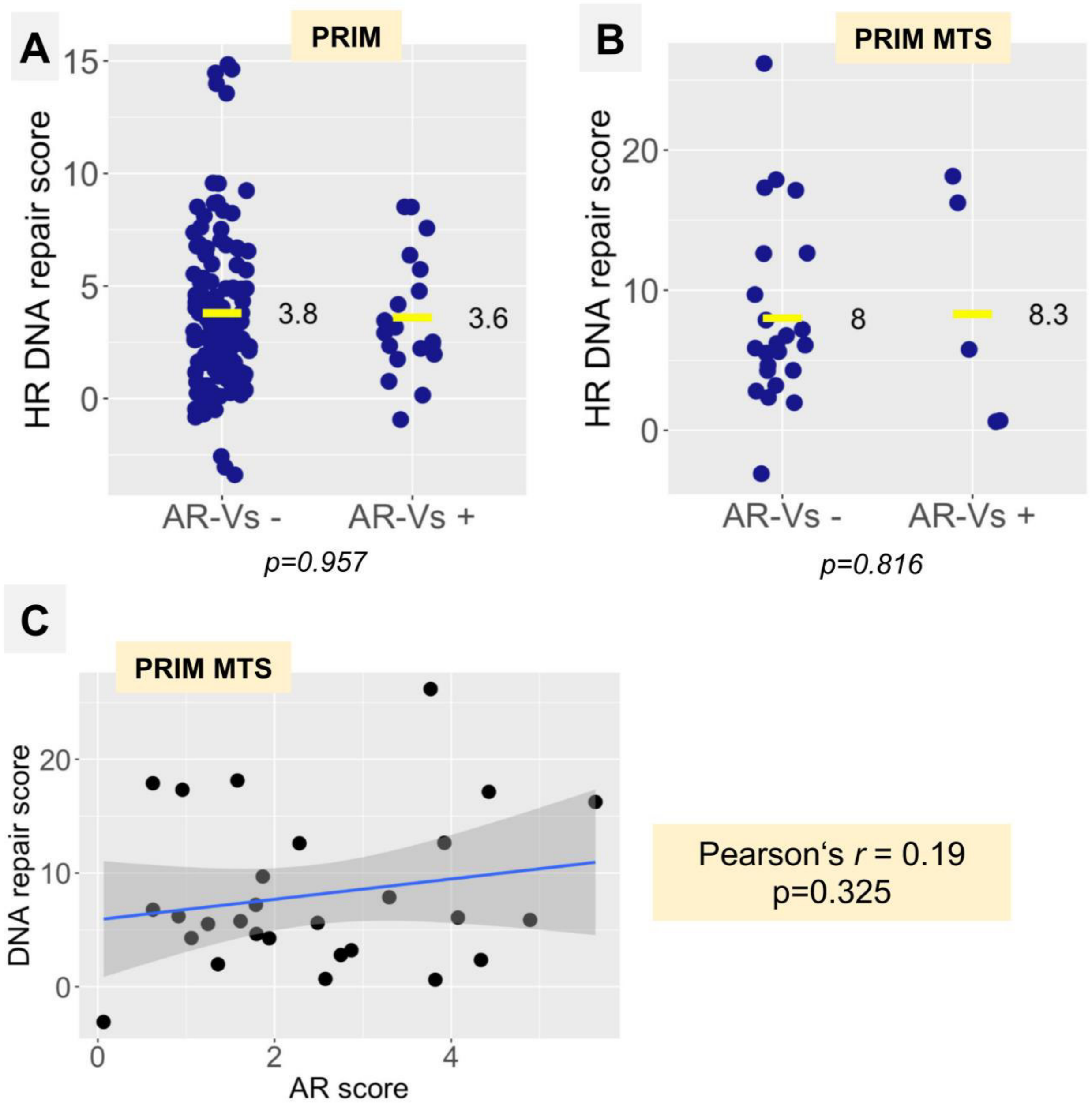

Figure S3: mRNA expression of 20 DNA repair genes in study groups and correlation of DNA repair and AR scores. *p*-levels were calculated using the Mann-Whitney U-test.

Figure S4: mRNA expression of 20 DNA repair genes in dependence on AR-V status (expression of any of four AR-V splice variants: V1, V3, V7, V9; light blue in all examined tissue types (PRIM, ADT, CRPC, PRIM MTS, BPH, NORM). Dark blue dots represent samples devoid of AR splice variants. p-values were calculated using the Mann-Whitney U-test. Groups were statistically significant: * vs PRIM group, ^ CRPC vs ADT group, # vs CTRL group, § vs PRIM MTS group.

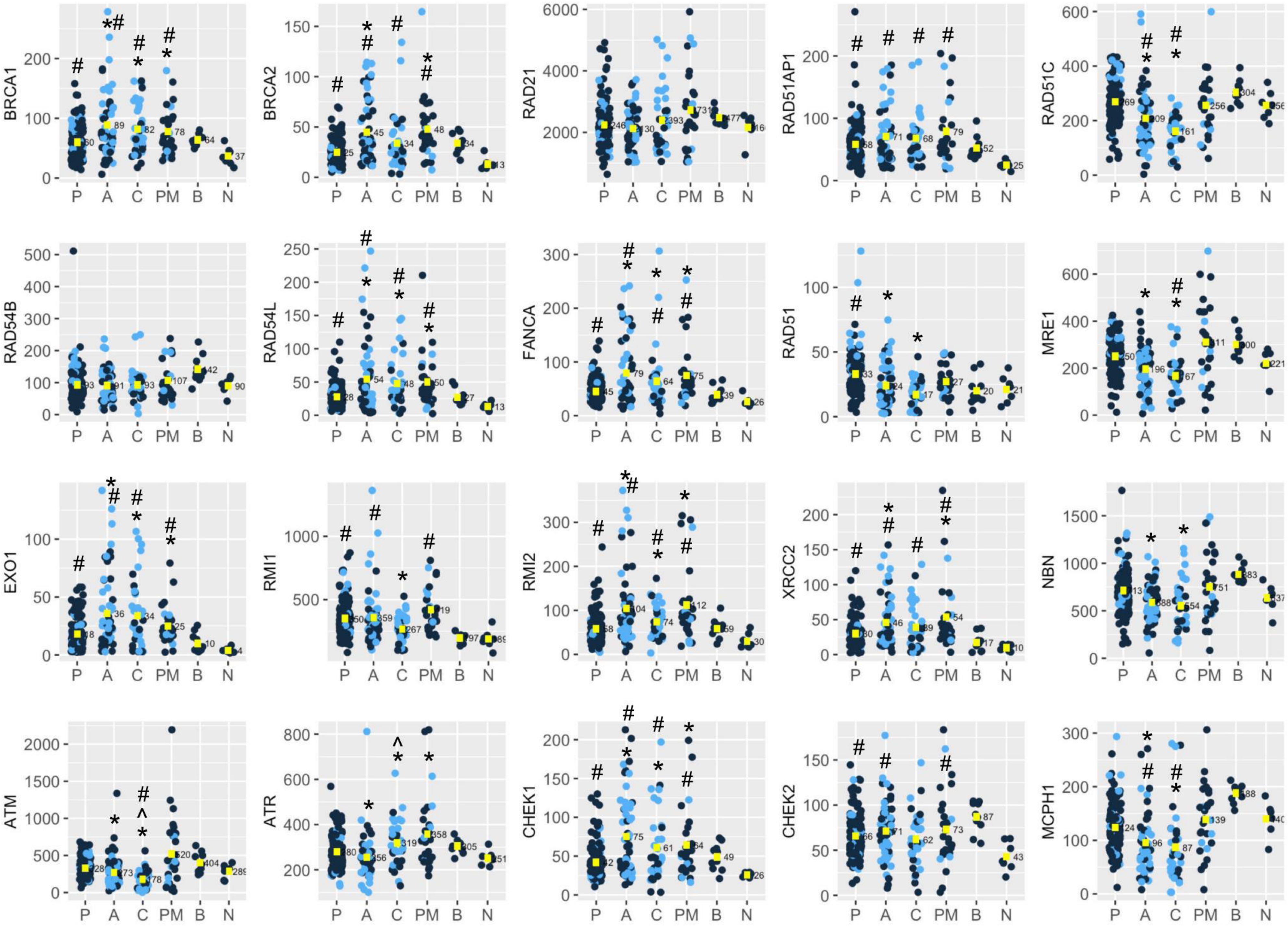

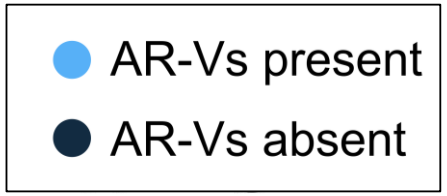

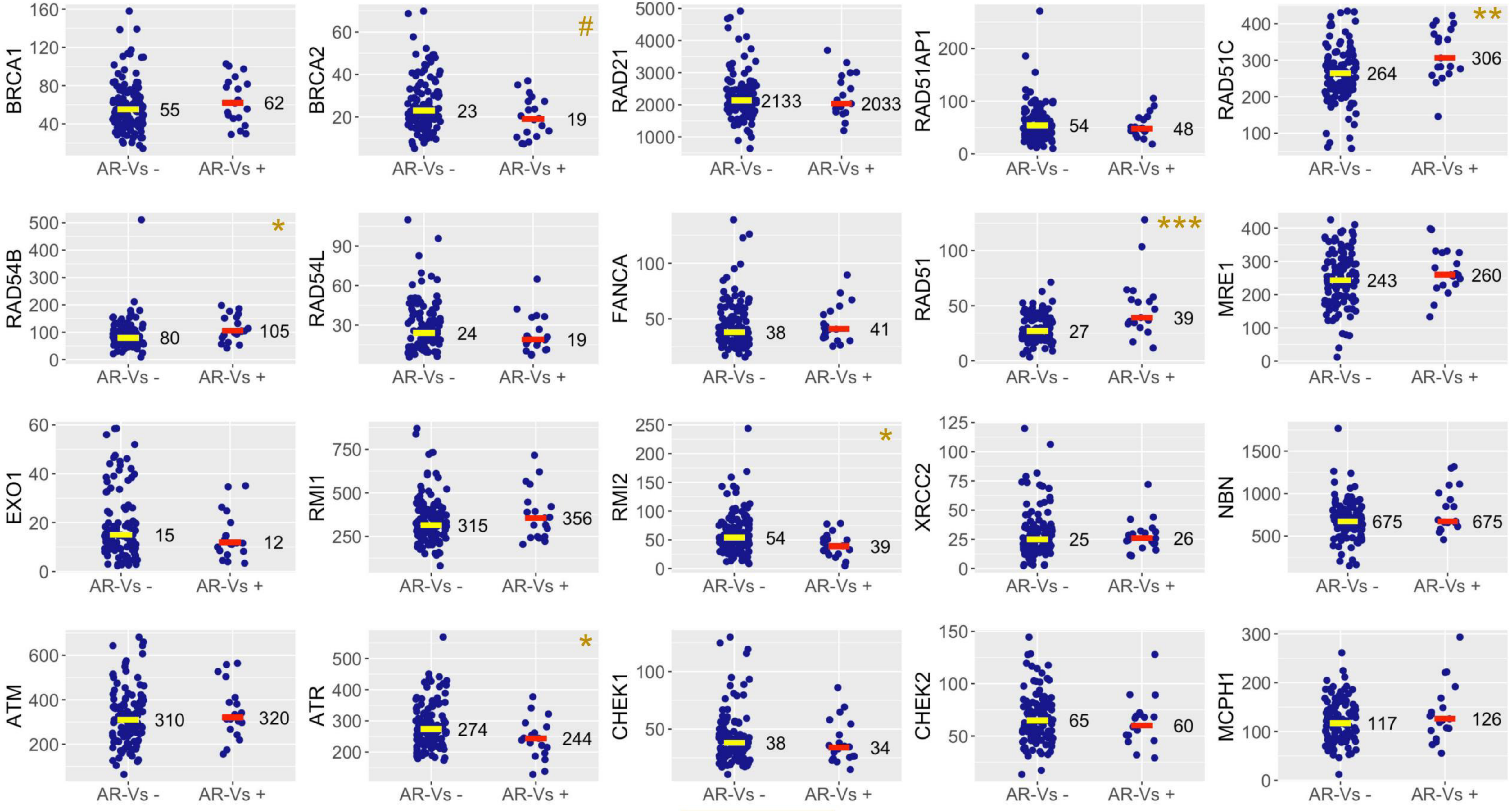

Figure S5: mRNA expression of 20 DNA repair genes in dependence on AR-V status in the PRIM group.
Expression of any of four AR-Vs: V1, V3, V7, V9. *p*-values were calculated using the Mann-Whitney U-test. * $p = 0.01 - 0.05$, ** $p = 0.01 - 0.001$, *** $p < 0.001$, # $p = 0.05 - 0.1$.

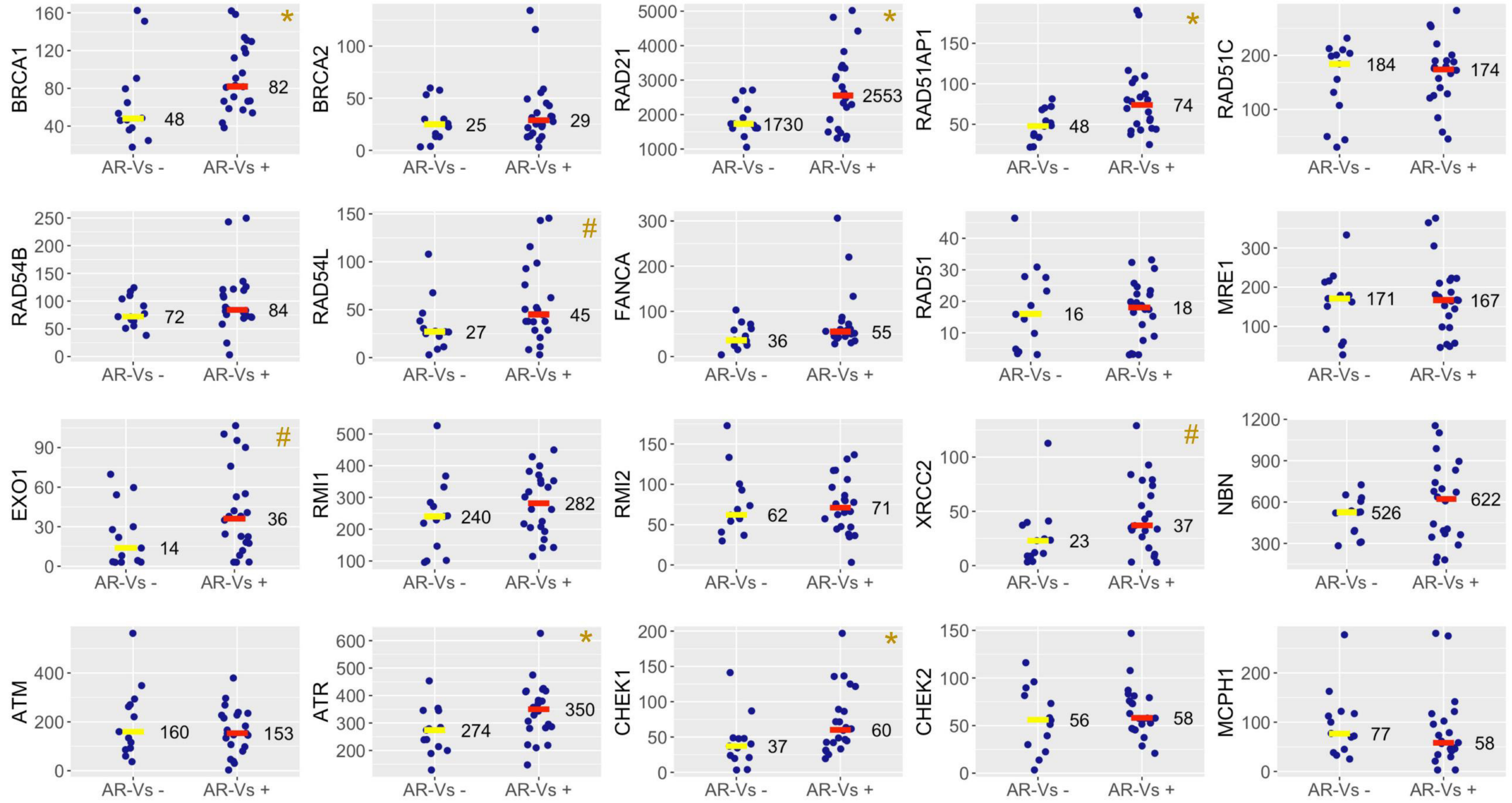

Figure S6: mRNA expression of 20 DNA repair genes in dependence on AR-V status in the CRPC group.

Expression of any of four AR-Vs: V1, V3, V7, V9. *p*-values were calculated using the Mann-Whitney U-test. * p = 0.01 - 0.05, # p = 0.05 - 0.1.

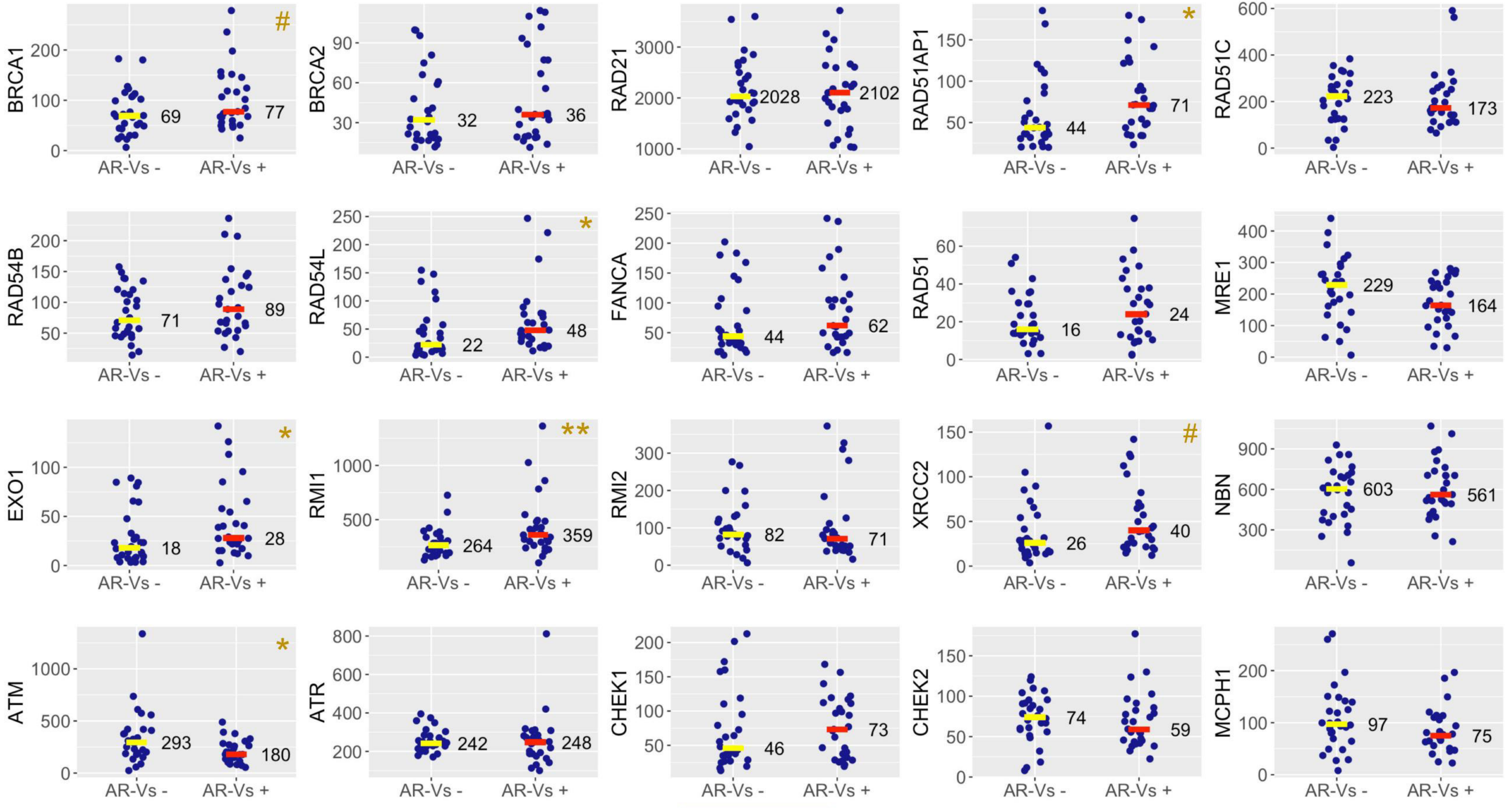

Figure S7: mRNA expression of 20 DNA repair genes in dependence on AR-V status in the ADT group.

Expression of any of four AR-Vs: V1, V3, V7, V9. *p*-values were calculated using the Mann-Whitney U-test. * p = 0.01 - 0.05, ** p = 0.01 - 0.001, # p = 0.05 - 0.1.

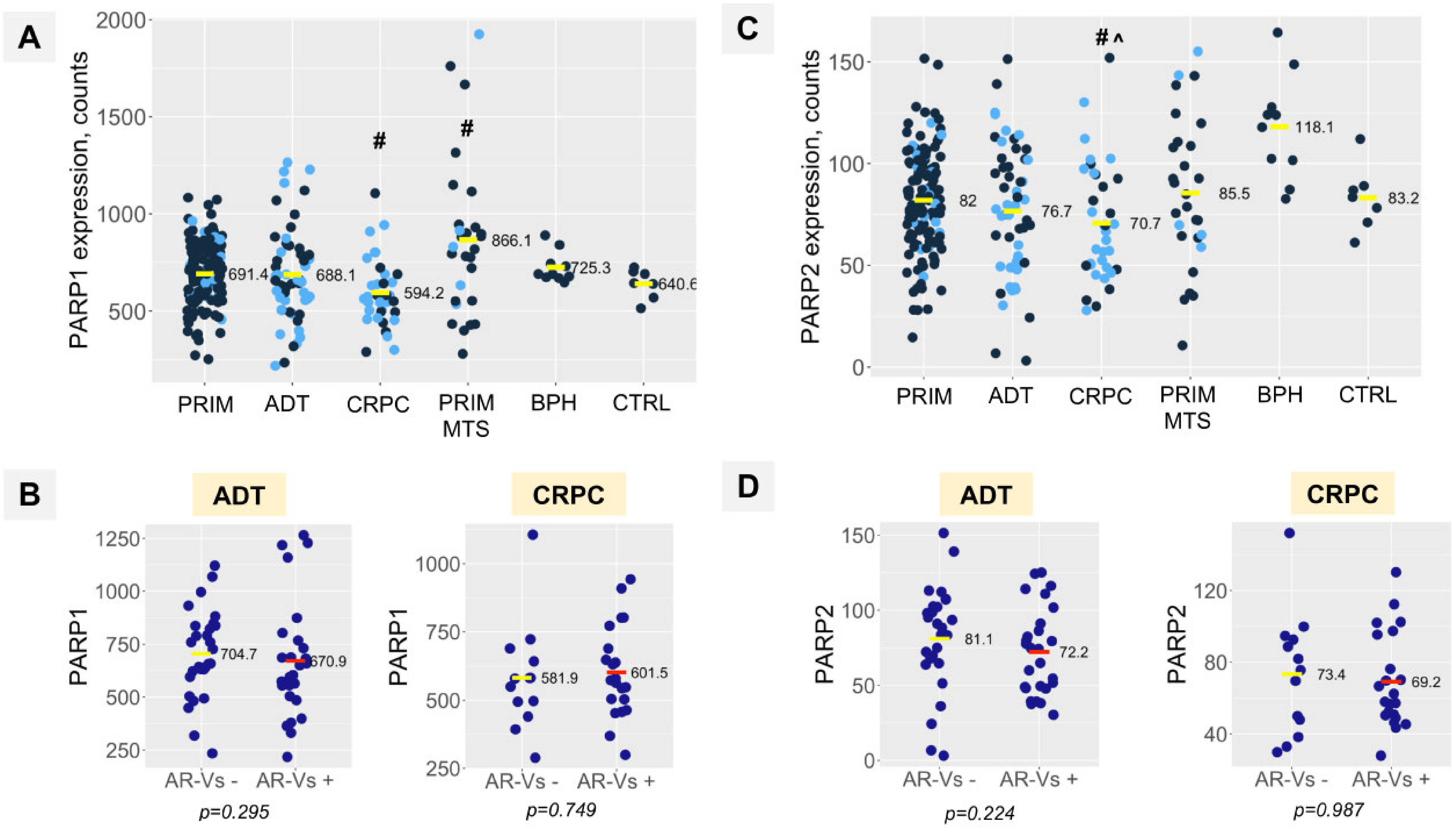
A
PARP1 expression, counts
2000
1500
1000
500
#
#
691.4
688.1
594.2
866.1
725.3
640.6
PRIM
ADT
CRPC
PRIM MTS
BPH
CTRL
C
PARP2 expression, counts
150
100
50
0
^
82
76.7
70.7
85.5
118.1
83.2
PRIM
ADT
CRPC
PRIM MTS
BPH
CTRL
B
ADT
PARP1
1250
1000
750
500
250
704.7
670.9
AR-Vs -
AR-Vs +
p=0.295
CRPC
PARP1
1000
750
500
250
581.9
601.5
AR-Vs -
AR-Vs +
p=0.749
D
ADT
PARP2
150
100
50
0
81.1
72.2
AR-Vs -
AR-Vs +
p=0.224
CRPC
PARP2
120
80
40
73.4
69.2
AR-Vs -
AR-Vs +
p=0.987

Figure S8: mRNA expression of PARP1 (A) and PARP2 (B) in study groups. Light blue points express any of AR-V splice variants (V1, V3, V7, V9), dark blue points do not express AR-V splice variants. PARP1 (**C**) and PARP2 (**D**) expression in ADT and CRPC groups in dependence on AR-V status (expression of any of four AR-V splice variants: V1, V3, V7, V9). p-levels were calculated using the Mann-Whitney U-test. Groups were statistically significant ($p<0.05$): ^ CRPC vs ADT group, $^{\#}$ vs CTRL group.

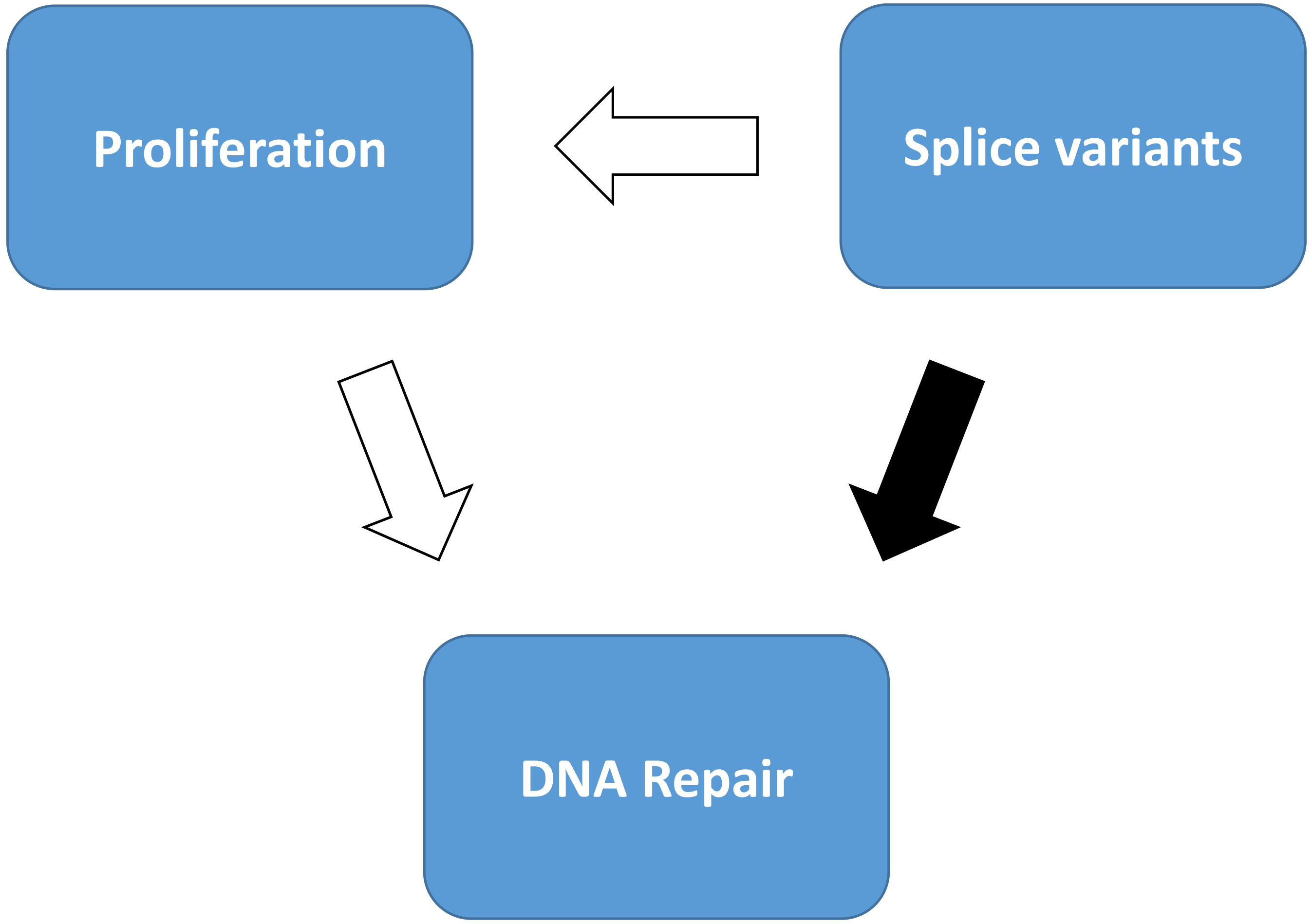

Figure S9: Separation of the impact of AR splice variants on proliferation and DNA repair using a linear regression model. Directed acyclic graph visualizing the relationships between AR splice variants, proliferation and DNA repair. Our linear regression model allowed to calculate an effect of AR splice variants on DNA repair gene regulation (black arrow) while accounting for the effect of proliferation on DNA repair (white arrow).

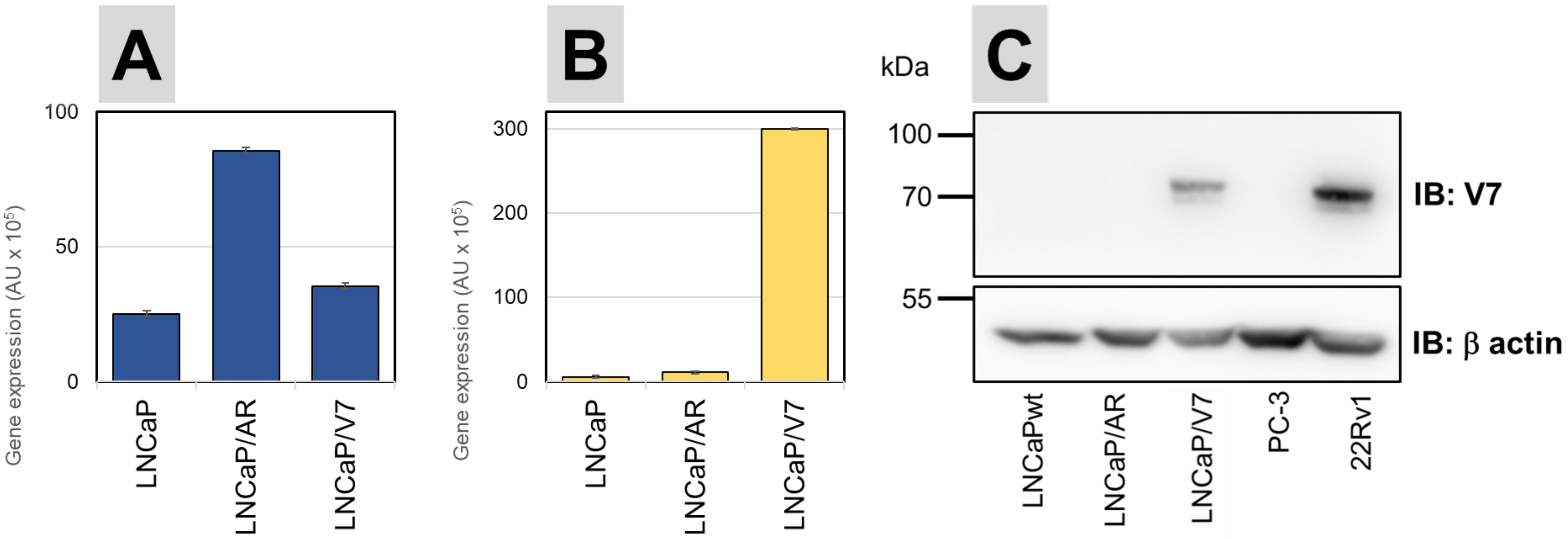

Figure S10: Expression of total androgen receptor and splice variant 7 in the LNCaP/AR *in vitro* tumor models. Parental LNCaPwt and derived cell lines overexpressing either full length AR (LNCaP/AR) or the AR-V7 splice variant (LNCaP/V7) were analyzed for total AR (A) or AR-V7 gene expression (B). AR-V7 protein was also detected by AR-V7 specific immunoblotting in LNCaP/V7. 22Rv1 cells, known to express AR-V7, served as control. None of the other cell lines expressed this splice variant, β actin was used as loading control (C). (AU – arbitrary units)

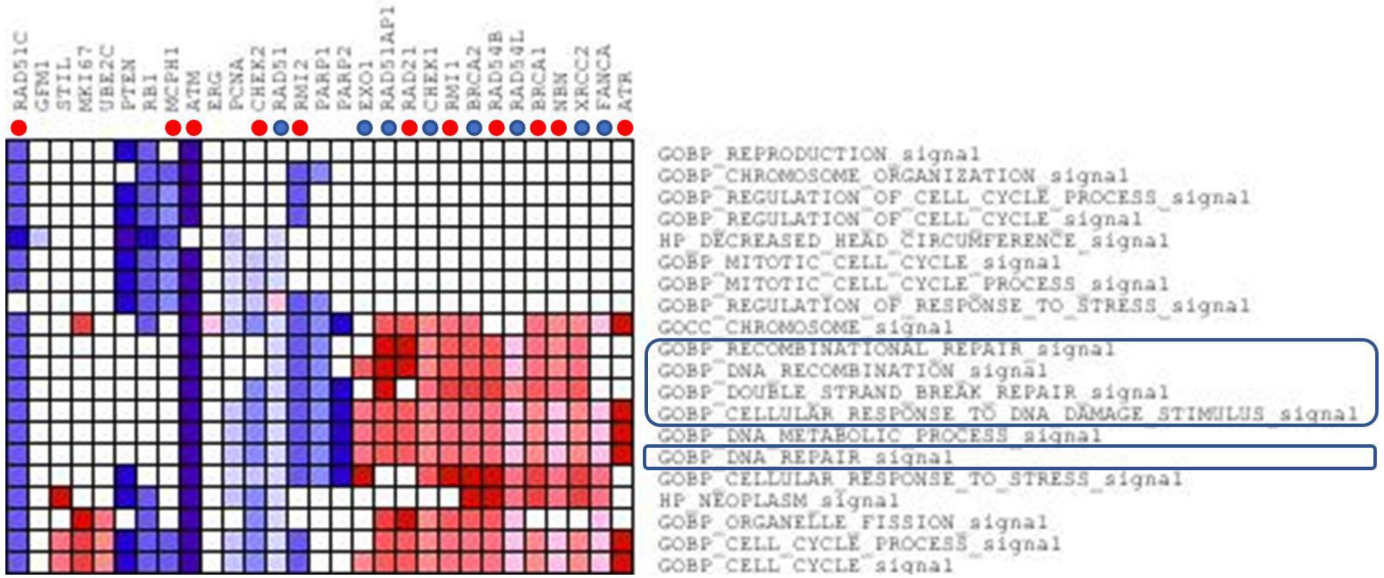

A

GO biological process complete	Homo sapiens - REFLIST (20589)	upload_1 (41)	upload_1 (expected)	upload_1 (over/under)	upload_1 (fold Enrichment)	upload_1 (raw P-value)	upload_1 (FDR)
DNA repair (GO:0006281)	508	22	1.01	+	21.75	1,05E-24	1.64E-20
double-strand break repair (GO:0006302)	203	17	0.4	+	42.05	1,93E-23	1.51E-19
double-strand break repair via homologous recombination (GO:0000724)	113	14	0.23	+	62.22	1,65E-21	8.60E-18
recombinational repair (GO:0000725)	117	14	0.23	+	60.09	2,59E-21	1.01E-17
cellular response to DNA damage stimulus (GO:0006974)	756	22	1.51	+	14.61	4,48E-21	1.40E-17
DNA metabolic process (GO:0006259)	794	22	1.58	+	13.91	1,25E-20	3.27E-17
DNA recombination (GO:0006310)	245	15	0.49	+	30.75	1,01E-18	2.27E-15
cell cycle (GO:0007049)	1249	23	2.49	+	9.25	8,44E-18	1.65E-14
cell cycle process (GO:0022402)	841	20	1.67	+	11.94	2,43E-17	4.24E-14

B

Figure S11: Gene ontology analysis of genes de-regulated in clinical samples reveals association with DNA repair. (A) Twenty pathways passed the threshold and are listed in the matrix. Pathways related to DNA repair (recombinational repair, DNA recombination, DSB repair, cellular response to DNA damage stimulus, DNA repair) are highlighted by blue frames and are associated with the majority of genes de-regulated in our analysis, but are not primarily involved in cell cycle pathways (right side). BRCA1 cluster genes are labeled by red dots, BRCA2 cluster genes by blue dots. (B) GO Panther hierarchical clustering ranks gene set analyzed in clinical samples most significantly with DNA repair pathways.

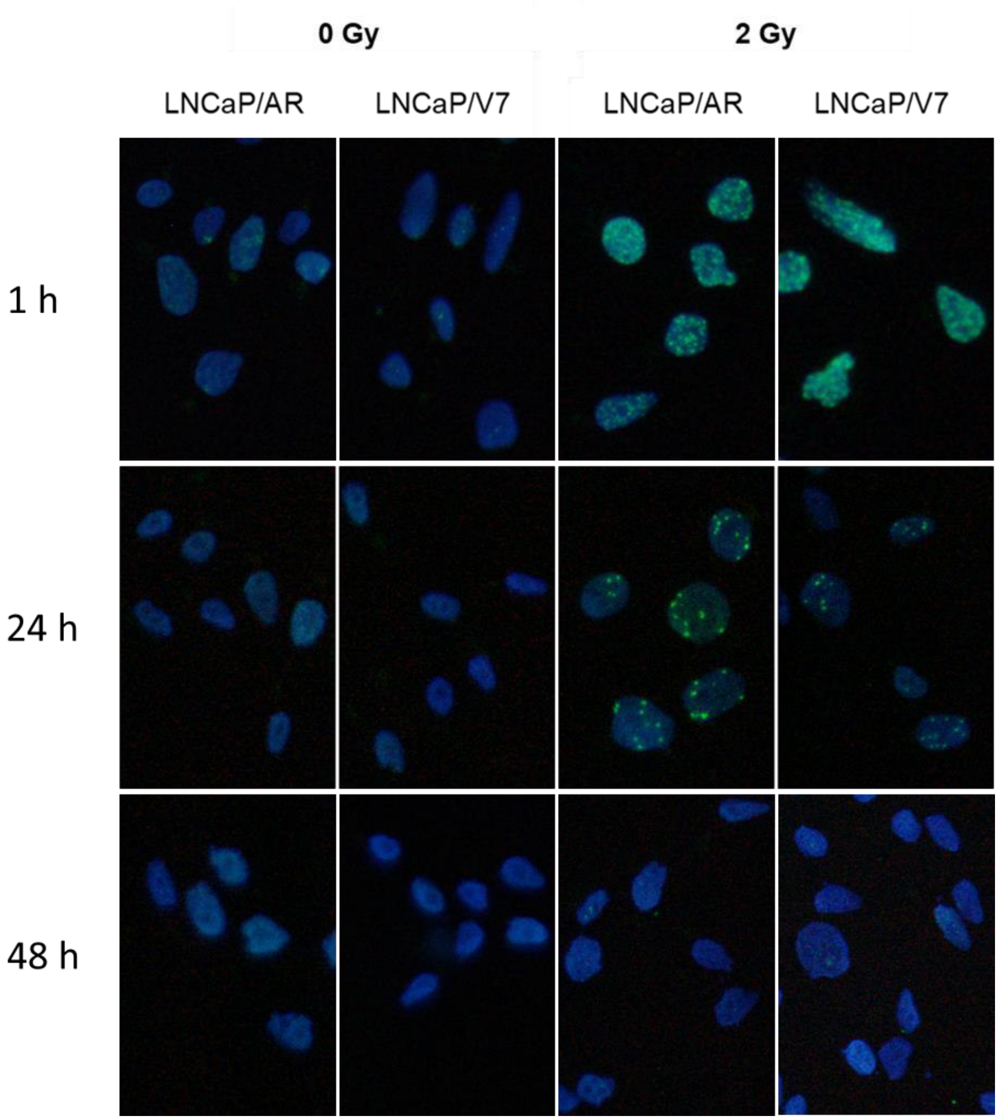

Figure S12: AR-V7 improves DNA repair after irradiation in an in vitro tumor model. Vizualization of gH2Ax foci. Nuclear counterstain with DAPI from left to right: LNCaP/AR (0 Gy), LNCaP/V7 (0 Gy), LNCaP/AR (2 Gy), LNCaP/V7 (2 Gy). Time points are 1 h, 24 h and 48 h after irradiation. Note the high number of foci immediately after irradiation compared to the almost complete loss of visible foci after two days.
